# NleC, a Type III Secretion Protease, Compromises NF-κB Activation by Targeting p65/RelA

**DOI:** 10.1371/journal.ppat.1001231

**Published:** 2010-12-16

**Authors:** Hilo Yen, Tadasuke Ooka, Atsushi Iguchi, Tetsuya Hayashi, Nakaba Sugimoto, Toru Tobe

**Affiliations:** 1 Department of Microbiology and Immunology, Graduate School of Medicine, Osaka University, Suita, Osaka, Japan; 2 Division of Microbiology, Department of Infectious Diseases, Faculty of Medicine, Frontier Science Research Center, University of Miyazaki, Miyazaki, Japan; 3 Interdisciplinary Research Organization, Frontier Science Research Center, University of Miyazaki, Miyazaki, Japan; 4 Division of Bioenvironmental Science, Frontier Science Research Center, University of Miyazaki, Miyazaki, Japan; Institut Pasteur, France

## Abstract

The NF-κB signaling pathway is central to the innate and adaptive immune responses. Upon their detection of pathogen-associated molecular patterns, Toll-like receptors on the cell surface initiate signal transduction and activate the NF-κB pathway, leading to the production of a wide array of inflammatory cytokines, in attempt to eradicate the invaders. As a countermeasure, pathogens have evolved ways to subvert and manipulate this system to their advantage. Enteropathogenic and enterohemorrhagic *Escherichia coli* (EPEC and EHEC) are closely related bacteria responsible for major food-borne diseases worldwide. Via a needle-like protein complex called the type three secretion system (T3SS), these pathogens deliver virulence factors directly to host cells and modify cellular functions, including by suppressing the inflammatory response. Using gain- and loss-of-function screenings, we identified two bacterial effectors, NleC and NleE, that down-regulate the NF-κB signal upon being injected into a host cell via the T3SS. A recent report showed that NleE inhibits NF-κB activation, although an NleE-deficient pathogen was still immune-suppressive, indicating that other anti-inflammatory effectors are involved. In agreement, our present results showed that NleC was also required to inhibit inflammation. We found that NleC is a zinc protease that disrupts NF-κB activation by the direct cleavage of NF-κB's p65 subunit in the cytoplasm, thereby decreasing the available p65 and reducing the total nuclear entry of active p65. More importantly, we showed that a mutant EPEC/EHEC lacking both NleC and NleE (*ΔnleC ΔnleE*) caused greater inflammatory response than bacteria carrying *ΔnleC* or *ΔnleE* alone. This effect was similar to that of a T3SS-defective mutant. In conclusion, we found that NleC is an anti-inflammatory bacterial zinc protease, and that the cooperative function of NleE and NleC disrupts the NF-κB pathway and accounts for most of the immune suppression caused by EHEC/EPEC.

## Author Summary

Enteropathogenic *Escherichia coli* (EPEC) and enterohemorrhagic *E. coli* (EHEC) cause food-borne diseases, including watery diarrhea or severe bloody diarrhea and life-threatening kidney disease (hemolytic uremic syndrome). Upon ingestion, EPEC/EHEC colonize the cells of the epithelial lining in the intestinal tract. In response, the affected cells initiate an immune response by secreting cytokines that attract immune cells. To prevent their early elimination by the host, these bacteria have developed strategies to prevent the host immune response. They do this by injecting bacterial effectors into the host cells to disrupt the NF-κB pathway, an essential effector of the host cell immune response. In the current study, we report the discovery of an NF-κB suppressive effector in EPEC/EHEC called NleC, and its novel mechanism. We found that NleC is a zinc protease that can digest p65, a critical component of the NF-κB pathway, thus dampening the host inflammatory response. NleE is another recently identified anti-inflammatory effector. We show here that an EPEC/EHEC mutant deficient in both NleC and NleE loses most of its ability to suppress the host inflammatory response. Our findings show how two different bacterial effectors can function in cooperation to modify the host immune response.

## Introduction

Enteropathogenic *Escherichia coli* (EPEC) and enterohemorrhagic *E. coli* (EHEC) are worldwide causative agents of illness and death [1]. EPEC causes infantile diarrhea, which is often lethal in developing countries, and EHEC is a frequent cause of bloody diarrhea and hemolytic uremic syndrome (HUS) even in developed countries [2]. These pathogens are often transmitted in contaminated food. Once they reach the human intestine, the bacteria multiply and colonize on the mucosal surface. These bacteria are also known as “attaching and effacing (A/E)” pathogens due to the histopathological lesions caused by the intestinal colonization [3], [4]. A/E lesions are characterized by localized damage to the intestinal microvilli and the rearrangement of host cytoskeletal proteins beneath the intimately attached bacterial colonies [5], [6].

The key virulence factors in A/E pathogens are encoded at the locus of enterocyte effacement (LEE). The LEE, which is required for the formation of A/E lesions during infection, encodes regulators, an adhesin (intimin), chaperones, a translocator, effector proteins, and type III secretion system (T3SS) components [6]. In particular, the T3SS, an organelle common to the A/E pathogens, is responsible for delivering bacterial effector proteins directly from the bacterial cytoplasm into the host cytoplasm, where they modify and disrupt host cell functions [6]. An isogenic mutant defective in the T3SS loses the ability to establish successful colonization on host cells, indicating that the T3SS is a major determinant of pathogenicity [7], [8]. In addition to the seven effector proteins encoded by the LEE, EPEC and EHEC possess a variety of effector proteins that are encoded elsewhere on the genome. The EHEC genome encodes more than 40 effector proteins [9]. and EPEC encodes at least 21 [10], [11]. Although the functions of several effector proteins have been reported, those of many others have yet to be determined [12].

The intestinal epithelium plays an important role in generating signals in response to pathogen infection to activate cells of the innate and acquired immune systems present in the underlying intestinal mucosa [13]. Mucosal inflammation is characterized by the coordinated expression and up-regulation of a specific set of gene products, including the cytokine and chemoattractant interleukin (IL)-8, and macrophage inflammatory protein 1α [14]. The NF-κB family proteins are key regulators of inflammatory genes; they are structurally similar transcription factors, and include c-Rel, RelB, p65 (RelA), p50 (p105 processed form), and p52 (p100 processed form) [15]. In unstimulated cells, homo- or heterodimers of NF-κBs are maintained in an inhibited state in the cytoplasm by their binding with IκB [16]. In an immune-challenged state or during infection, stimulated Toll-like receptors (TLRs) initiate a signaling cascade that results in activation of the IκB kinase (IKK) complex [17], [18]. The IKK complex phosphorylates and ubiquitinates IκB, marking it for degradation, which frees the NF-κB dimmers [15], [19]. The dimers then enter the nucleus and promote the transcription of genes for inflammatory proteins such as IL-8, IL-1β, and TNF-α [20].

Over the course of EPEC or EHEC infection, the balance between the pro-inflammatory bacterial extracellular components and the proposed anti-inflammatory effector proteins shapes the outcome of the host immune responses [21], [22], [23]. At the early stage of infection, the NF-κB-mediated inflammatory response is activated by T3SS-independent mechanisms; at later stages, the response is repressed in a T3SS-dependent manner [18], [19]. Several studies have indicated that flagellin is a major pro-inflammatory mediator [20], [21], [22]; the detection of flagellin by TLR-5 signals the activation of NF-κB to cause inflammation. On the other hand, in differentiated Caco-2 cells infected by EPEC, subsequent stimulation with TNF-α does not activate or cause the nuclear translocation of NF-κB [18], and this inhibition is independent of the LEE-encoded effectors [19]. Together, these observations suggest that anti-inflammatory activity may be mediated by non-LEE-encoded effector(s).

Recently, NleH and NleE have been shown to suppress host NF-κB activation independently, through different mechanisms [24], [25], [26]. NleH inhibits the translocation of ribosomal protein S3 (RPS3) into the nucleus, by binding to it in the cytoplasm, thereby reducing the activation of NF-κB/RPS3-dependent promoters [24]. A report on NleE elegantly demonstrated that NleE acts by interfering with the activation of the IKK complex, thereby maintaining the NF-κB dimers in an inhibited state [25]. However, unlike the T3SS-defective EPEC, the host immune response shows residual repression when infected with the Δ*nleH* or Δ*nleE* mutant of EPEC. This suggests the existence of an as-yet-unidentified non-LEE effector protein(s).

In the present study, by using an artificially created, reconstituted TOB02 strain (EPI300/LEE+BFP) that possesses the LEE, *bfp* operon, and *perABC* regulators of EPEC, and a series of EPEC deletion mutants, we have identified NleC as a novel effector that suppresses NF-κB activation. We determined that NleC acts by directly cleaving the NF-κB subunit p65 to a form that is degraded by proteasomes. We also showed that an EPEC compound mutant lacking both NleC and NleE was less effective at suppressing NF-κB activation than EPEC deficient in either gene alone (Δ*nleC* or Δ*nleE*). Furthermore, infection with the double mutant of EHEC or EPEC leaves the host response toward inflammatory stimulants nearly intact, at the level seen with a T3SS-defective strain.

## Results

### Characterization of a reconstituted bacterium possessing the LEE and *bfp* operon

**Figure 1:**
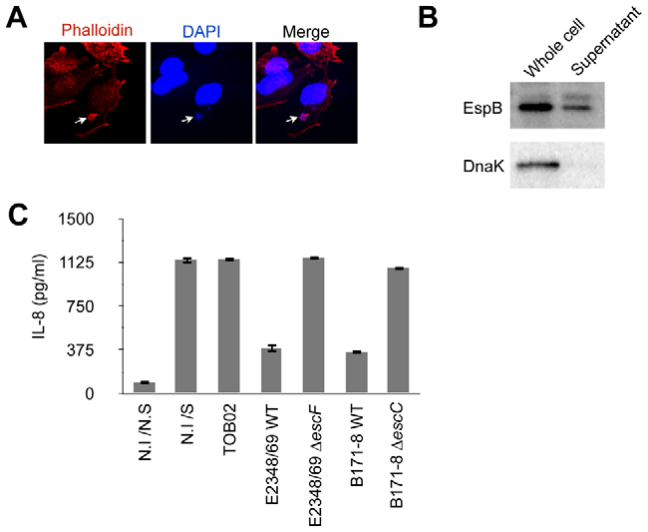
TOB02 is a functional tool for studying the effects of individual effectors. (A) Induction of actin polymerization by infection with TOB02. HeLa cells were infected with TOB02 for 3 hours. The cells were then fixed with 4% PFA and stained with Rhodamine-Phalloidin (red) and DAPI (blue). Arrow indicates the location of bacterial attachment. (B) Secretion of LEE-encoded virulence factor by TOB02. Overnight-grown TOB02 was inoculated and cultured in fresh DMEM until O.D_600_ ∼1.0. The cell pellet (Whole cell) and culture supernatant (Supernatant) were collected and analyzed with anti-EspB and anti-DnaK antibodies. (C) Cellular response to infection with reconstituted strains. HeLa cells were infected with TOB02, E2348/69 WT (EPEC wild type), E2348/69 Δ*escF* (ΔT3SS), B171-8 WT (EPEC wild type), and B171-8 Δ*escC* (ΔT3SS) for 3 hours. Then cells were rinsed and incubated in fresh DMEM containing 0.1 mg/ml gentamicin and heat-killed bacteria (HKE) (10^8^/ml). Cells were further cultured for 8 hours, and the culture supernatants were analyzed for IL-8 by ELISA. Negative and positive controls were non-infected/non-stimulated (N.I/N.S) and non-infected/stimulated (N.I/S) cells, respectively.

To evaluate the effect of individual non-LEE effectors on host-cell immune responses, we employed a reconstituted *Escherichia coil* K12 strain that carries two plasmids, one harboring the full LEE locus and the other bearing a *bfp* operon for the bundle-forming pilli and the *perABC* regulatory genes of EPEC B171-8 (hereinafter referred to as TOB02) (Fig. S1). The LEE locus encodes the injectisome of the type III secretion (T3S) apparatus and essential virulence factors such as Tir and Intimin, which are necessary for intimate attachment, a hallmark of the EPEC/EHEC adherence [2]. To confirm that TOB02 was capable of colonization and establishing intimate attachment, we infected HeLa cells with it and looked for the reorganization of F-actin beneath the attached bacteria, a characteristic result of the intimate attachment that is dependent on virulence factors encoded by LEE. Following the infection, the cells were stained with DAPI and Rhodamine-phalloidin. Similar to infections of EPEC, microcolonies formed within 30 min of the infection, and an intense accumulation of F-actin was observed beneath the bacterial colonies (Fig. 1A). To confirm that the reconstituted TOB02 bacterium expressed a functional T3S system (T3SS), we assayed the secretion of a LEE-encoded protein, EspB, which is secreted through the T3SS [25]. We probed the culture supernatant of the TOB02 cells with an anti-EspB antibody, and detected EspB in an immunoblot (Fig. 1B). In addition, we also compared the amount of Tir translocated into host cells between the native EPEC and TOB02 strains (Fig. S2). Taken together, these results indicate that the T3SS of TOB02 is functional and that this strain can establish an intimate attachment to epithelial cells.

We next analyzed the inflammatory response of host cells upon TOB02 infection. As reported previously, the detection of PAMPs (pathogen-associated molecular patterns) by TLRs on the cell surface triggers the inflammatory response, which is suppressed by EPEC/EHEC during the early stage of colonization. Because T3SS-defective EPEC/EHEC cannot suppress the host immune response, the suppression of the host inflammatory response has been proposed as a T3SS-dependent phenomenon [22], [23], and therefore effectors on the LEE locus or non-LEE loci have been predicted to downregulate the host immune responses following the T3SS injection [22], [23]. To compare the response between cells infected with TOB02 and wild-type EPEC, we infected HeLa cells with TOB02 or with wild-type EPEC strains E2348/69 and B171-8 and their ΔT3SS mutants (E2348/69 Δ*escF* and B171-8 Δ*escC*). Following the infection, the cells were stimulated with heat-killed *E. coli* (HKE), and the amount of secreted IL-8 was measured. We found that cells infected with TOB02 released a large amount of IL-8 (Fig. 1C). Although TOB02 possesses the LEE-encoded effectors, the lack of a marked reduction in the inflammatory response of the TOB02-infected cells indicated that effectors residing on the LEE locus may not be very important in the host inflammatory suppression. Therefore, we used the TOB02 strain to determine which effectors on non-LEE loci could cause reduced immune responses upon infection.

### NleC reduced the inflammatory response by inhibiting NF-κB signaling

**Figure 2:**
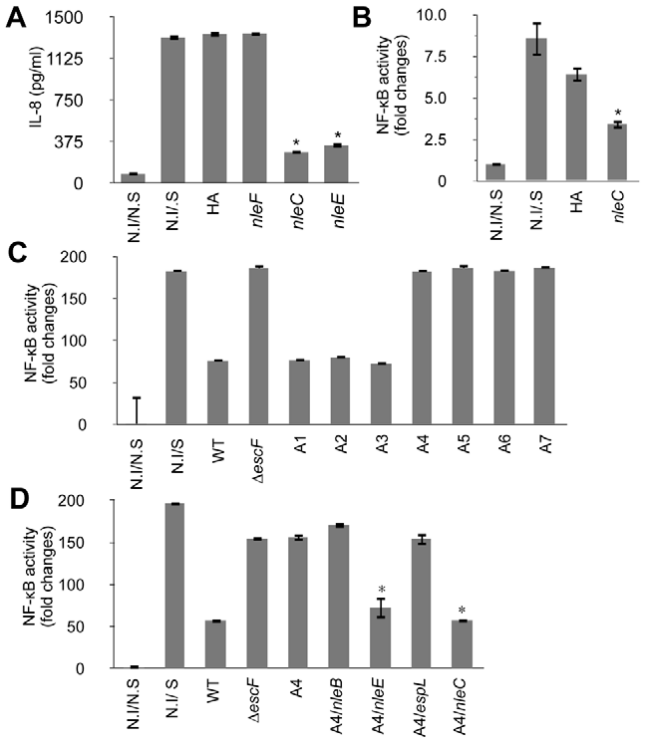
NleC can inhibit NF-κB activation. Two days prior to infection, HeLa cells were transfected with NF-κB and Luciferase reporter plasmids. On the day of infection, HeLa cells were infected with various bacterial strains for 3 hours. The medium was replaced with fresh DMEM containing gentamicin and HKE. The cells were further cultured for 8 hours, then the IL-8 or reporter activities of NF-κB and Luciferase were determined. The positive and negative controls were non-infected/non-stimulated (N.I/N.S) and non-infected/stimulated (N.I/S) HeLa cells. In the reporter assays, the NF-κB activity was normalized to the luciferase activity. All experiments were performed in triplicate and repeated three times. Representative results from the repeated experiments are shown. Significance was calculated by student's t-test, where p<0.05 was considered significant. (A) IL-8 secretion by cells infected with an *nleC-* or *nleE-*expressing strain. Cells were infected with TOB02/HA (HA), TOB02/*nleF*-HA (*nleF*), TOB02/*nleC*-HA (*nleC*), or TOB02/*nleE*-HA (*nleE*). IL-8 was analyzed by ELISA. (* p<0.05, compared to HA). (B) NF-κB activation in cells infected with an *nleC-*expressing strain. Cells were infected with TOB02/HA or TOB02/*nleC*-HA (*nleC*). (* p<0.05, compared to HA) (C) NF-κB activity in cells infected with EPEC mutants. For infection, wild-type EPEC (WT), a ΔT3SS mutant (Δ*escF*), and a series of isogenic mutants was used: A1 (deletion of IE2 region), A2 (A1 plus PP2 region deletion), A3 (A2 plus PP4 region deletion), A4 (A3 plus IE6 region deletion), A5 (A4 plus PP6 region deletion), A6 (A5 plus IE5 region deletion), and A7 (A6 plus *espG* deletion). (* p<0.05, compared to WT) (D) NF-κB activity in cells infected with an EPEC mutant expressing non-LEE effectors. Cells were infected with wild-type EPEC (WT), ΔT3SS mutant (Δ*escF*), A4, or A4 harboring a plasmid with *nleB* (A4/*nleB*), *nleE* (A4/*nleE*), *espL* (A4/*espL*), or *nleC* (A4/*nleC*). (* p<0.05, compared to A4).

Since EPEC and EHEC are closely related pathogens that suppress host immune responses [22], [23], we postulated that the T3SS-dependent anti-inflammatory effector proteins might be well conserved between these pathogens. We therefore compared the repertoires of the T3SS-dependent non-LEE effector proteins of EPEC (E2348/69 and B171-8) [10], [11] and EHEC (O157:H7 Sakai, O26, O111, O103) [9], [27], [28], and established strains of TOB02 carrying each of these shared non-LEE-effector genes (*espF*, *espG*, *espJ*, *espK*, *espO*, *espZ*, *Map*, *nleA*, *nleB*, *nleF*, *nleH1*, *nleG*, *nleC*, *nleE*, and *espH*). HeLa cells were infected with each strain followed by stimulation with HKE, and the IL-8 released into the medium was measured. Strains that caused a significantly lower secretion of IL-8 in comparison with the empty-vector control (TOB02/HA) were considered effective. Using this method, we identified NleC and NleE as effectors that could greatly decrease IL-8 secretion by the host cells (Fig. 2A).

Recently, Nadler *et al*. and Newton *et al*. reported that NleE blocks NF-κB signaling by inhibiting TAK1/IKK activation [25], [26]. Thus, the identification of NleE using the TOB02 system supported the validity of our screening method. We therefore focused on analyzing NleC. We first confirmed the secretion of NleC-HA by TOB02/*nleC*-HA (Fig. S3). Because IL-8 is a downstream target of the activated NF-κB pathway, we used an NF-κB reporter assay to verify that TOB02/*nleC*-HA could interfere with the NF-κB signaling cascade. HeLa cells transfected with reporter plasmids that harbored a NF-κB-regulated SEAP gene and a constitutively active luciferase gene, were infected with either TOB02/HA or TOB02/*nleC*-HA. After 3 hours of infection, the cells were further stimulated with HKE and assayed for the NF-κB reporter activity. As shown in Figure 2B, the NF-κB activity in the cells infected with TOB02/*nleC*-HA was significantly lower than in the cells infected with TOB02/HA. These results suggested that NleC suppressed the NF-κB activation, which led to the reduced secretion of IL-8 by the host cells.

### EPEC mutants lacking multiple effector gene loci lost most of their suppression activity

In our effort to screen for potential immune-suppressive non-LEE effectors, we devised another system using a series of EPEC mutants. These mutants, designated TOE-A1 to -A7, were designed to lose a cluster of effector genes in each specific horizontally transferred element (IE: integrative element, and PP: prophage) in a step-wise and additive fashion (Table S1). We used this approach to identify effectors with similar anti-inflammatory effects. We infected HeLa cells with these mutants, and the activity of the NF-κB reporter was determined after stimulating the cells with HKE. In this experiment, TOE-A4 lost most of the host immune suppression activity (Fig. 2C). TOE-A4 was derived from TOE-A3 by the deletion of a cluster of effector genes in IE6, which contains three effector genes, *nleE*, *nleB1*, and *espL*. Thus, TOE-A4 lacked nine effector genes (*nleB2*, *nleH1*, *espJ*, *nleG*, *nleC*, *nleD*, *nleE*, *nleB1*, and *espL*: Table S1). Since NleE is required but insufficient for the full repression of NF-κB activation by EPEC, additional effectors were predicted to be involved [25]. Furthermore, host cells infected with TOE-A5 to -A7 did not show a significantly greater recovery of their NF-κB activity than cells infected with TOE-A4 or Δ*escF* mutant, suggesting that none of the non-LEE effector proteins besides the ones missing from TOE-A4 contributed significantly to the disruption of the NF-κB signaling cascade. In another words, the full de-repression of the NF-κB activity by EPEC can be attributed to the combined loss of NleE and one or more of the protein(s) missing from the TOE-A4 mutant.

From our primary screening with the TOB02 system, we ruled out *nleH1*, *espJ*, *nleG*, *nleD*, *espL*, or *nleB*, because they were unable to suppress the host inflammatory response to HKE (data not shown). Therefore, we examined EPEC TOE-A4 harboring an *nleC-*expressing plasmid for the ability to suppress NF-κB activity. We found that TOE-A4/*nleC* restored the suppression of the host NF-κB activity to a level near that achieved with TOE-A4/*nleE* or wild type (Fig. 2D). Taken together, our results strongly indicated that NleC is an immune suppressor with activity comparable to NleE in a complementary setting that may act in parallel to or be redundant with NleE.

### Repression of NF-κB activation by NleC was independent of other bacterial factors

**Figure 3:**
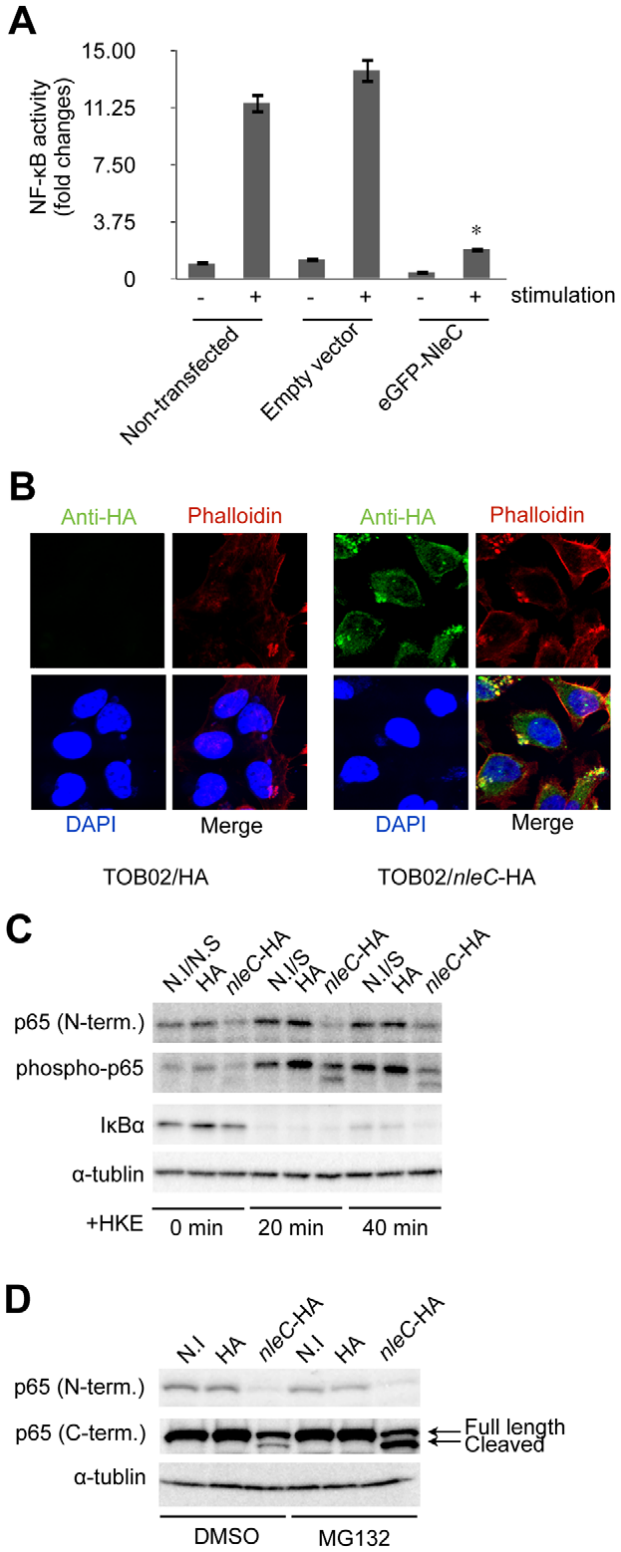
NleC interferes with NF-κB activation by downregulating p65. (A) NF-κB activity in cells transfected with *nleC*. HeLa cells were all transfected with NF-κB and luciferase reporter plasmids. In addition, either eGFP plasmid (Empty vector) or eGFP-NleC-expressing plasmid was introduced into the cells. “Non-transfected” indicates cells transfected with only the reporter plasmid. Two days post-transfection, the cells were stimulated with HKE. The culture medium and cell lysates were then analyzed for NF-κB SEAP and Luciferase activity. (* p<0.05, compared to stimulated Empty Vector). (B) Localization of NleC in infected cells. HeLa cells were infected with either TOB02/HA or TOB02/*nleC*-HA. After 3 hours of infection, cells were washed and fixed with 4% PFA, then stained with anti-HA (FITC), phalloidin (red), and DAPI (blue). (C) Decrease in the IκBα and p65 proteins in cells infected with the *nleC-*expressing strain. HeLa cells were infected with TOB02/HA (HA) or TOB02/*nleC*-HA (*nleC*-HA). After 3 hours of infection, the cells were rinsed and incubated in fresh DMEM containing 0.1 mg/ml gentamicin and a 1/10 volume of HKE. HKE stimulation proceeded for 20 and 40 min, and cells were collected for western blot analysis. Cell extracts were probed with anti-p65 (N-term.), anti-phospho-p65, anti-IκBα, and anti-α-tubulin (as a loading control) antibodies. Non-infected/Non-stimulated cells at 0 min. (N.I/N.S at 0 min.) served as a negative control. Non-infected/stimulated (N.I/S) cells at 20 and 40 min were positive controls. (D) Appearance of a cleaved fragment of p65 in cells infected with the NleC-expressing strain. Two hours prior to infection, HeLa cells were pretreated with either DMSO or MG132 (5 µM). The cells were infected with TOB02/HA (HA) or TOB02/*nleC*-HA (*nleC*-HA) for 3 hours. The cells were then rinsed and lysates were prepared for analysis. Anti-p65 antibodies specific to either the N- or C- terminal regions were used. Non-infected (N.I) cells served as a negative control.

To determine whether the function of NleC required other bacterial co-factors, we constructed a mammalian expression vector bearing a fusion protein, eGFP-NleC, and introduced it into HeLa cells together with the NF-κB reporter plasmid. After stimulating the cells with HKE, the reporter activities were determined. As shown in Figure 3A, whereas the non-transfected (NF-κB reporter plasmid only) and empty-vector controls showed high NF-κB activity in response to the HKE stimulation, the eGFP-NleC-expressing HeLa cells showed markedly lower NF-κB activation. Taken together, these results suggested that NleC is a negative regulator of the host NF-κB signaling pathway, and that it does not require bacterial co-factors for its function.

The NF-κB signaling cascade is divided mainly into cytoplasmic and nuclear portions [15]. To elucidate where NleC functions to interfere with this cascade, we examined its localization after being injected into host cells via T3SS. After 3 hours of infection with TOB02/*nleC*-HA, the cells were fixed and stained with anti-HA/anti-mouse IgG-FITC, Rhodamine-Phalloidin, and DAPI. Most of the NleC-HA was apparently in the cytoplasm, although the strongest signals were observed beneath the microcolonies (Fig. 3B). We also found that NleC-HA showed the same distribution pattern in host cells infected with wild-type EHEC, via the endogenous T3SS (Fig. S4). These results suggested that NleC targets one or more cytoplasmic factors in the host NF-κB pathway.

### NleC decreased the p65 protein level in the host cell

Since NleC was determined to be predominantly cytoplasmic, and since various upstream signals converge to modify the IκBα/p65/p50 complex, which serves as a signaling hub, we decided to first examine changes that might occur at this complex. To do this, HeLa cells were infected with either TOB02/HA or TOB02/*nleC*-HA bacteria. After 3 hours of infection, the HeLa cells were further stimulated with HKE for 20 and 40 min, before being collected for western blot analysis. Using antibodies specific to the N-terminal region of p65 (p65 N-term), phospho-p65, and IκBα, we detected a significant down-regulation of p65 in cells infected with TOB02/*nleC*-HA (Fig. 3C). Compared to the level in cells infected with TOB02/HA, the amount of total p65 as well as the phospho- (active) p65 was lower in the TOB02/*nleC*-HA-infected cells even before the HKE stimulation. These results suggested that NleC may either directly or indirectly target p65 to decrease NF-κB signaling.

Based on this result, we speculated that NleC promotes the degradation of p65 in the cytoplasm. The ubiquitination-proteasome pathway is involved in selective protein decomposition, and several studies have shown that p65 is poly-ubiquitinated and targeted for proteasomal degradation [29], [30], [31], [32]. We therefore tested whether MG132, a reversible inhibitor of proteasomes, could prevent the p65 degradation in the presence of NleC. HeLa cells pretreated with either DMSO or MG132 (5 µM) were infected with TOB02/HA or TOB02/*nleC*-HA for 3 hours. As shown in Figure 3D, using the p65 N-terminal specific antibody, we found that the total p65 decreased substantially regardless of DMSO or MG132 treatment in the TOB02/*nleC*-HA infected cells.

Next, we used a C-terminal-specific antibody to detect p65, which at first glance confirmed the reduction of the protein. However, upon closer examination, we noticed a smaller fragment in addition to the full-length p65 that was specific to the TOB02/*nleC*-HA-infected cells (Fig. 3D; middle panel). Moreover, treatment with MG132 or lactacystin resulted in an accumulation of this smaller fragment (Fig. S5). This observation suggested that NleC may be directly or indirectly involved in generating this cleaved product, which is targeted for proteasomal degradation. Thus, our findings strongly suggest that NleC injection could trigger the reduction of NF-κB activity by decreasing the amount of p65 in infected cells.

### NleC directly cleaved p65 at its N-terminal domain

Since the smaller p65 (hereafter referred to as p65-C) could only be detected by the C-terminal-specific antibody, and the size difference between the full-length and cleaved form differed by only several kiloDaltons, we predicted that the cleavage site would be located at N-terminus. To determine the actual cleavage site, we extracted p65-C and analyzed its amino acid sequence from the N-terminal end. We found that the first four amino acids matched the 11th to 14th residues of the full length p65. Therefore, we concluded that the cleavage occurs between the 10th and 11th amino acid of p65. Coiras *et al*. reported that activated caspase-3 can mediate the cleavage of p65 at its N-terminal region to generate a C-fragment in non-apoptotic T-lymphocytes [33]. To examine the possible involvement of caspase-3 in the cleavage of p65 in cells infected with *nleC*-expressing bacteria, we pretreated HeLa cells with either DMSO or z-VAD-fmk (a pan-inhibitor of caspase). However, the inhibition of caspase-3 activity with z-VAD-fmk did not affect the generation of p65-C (Fig. S6).

**Figure 4:**
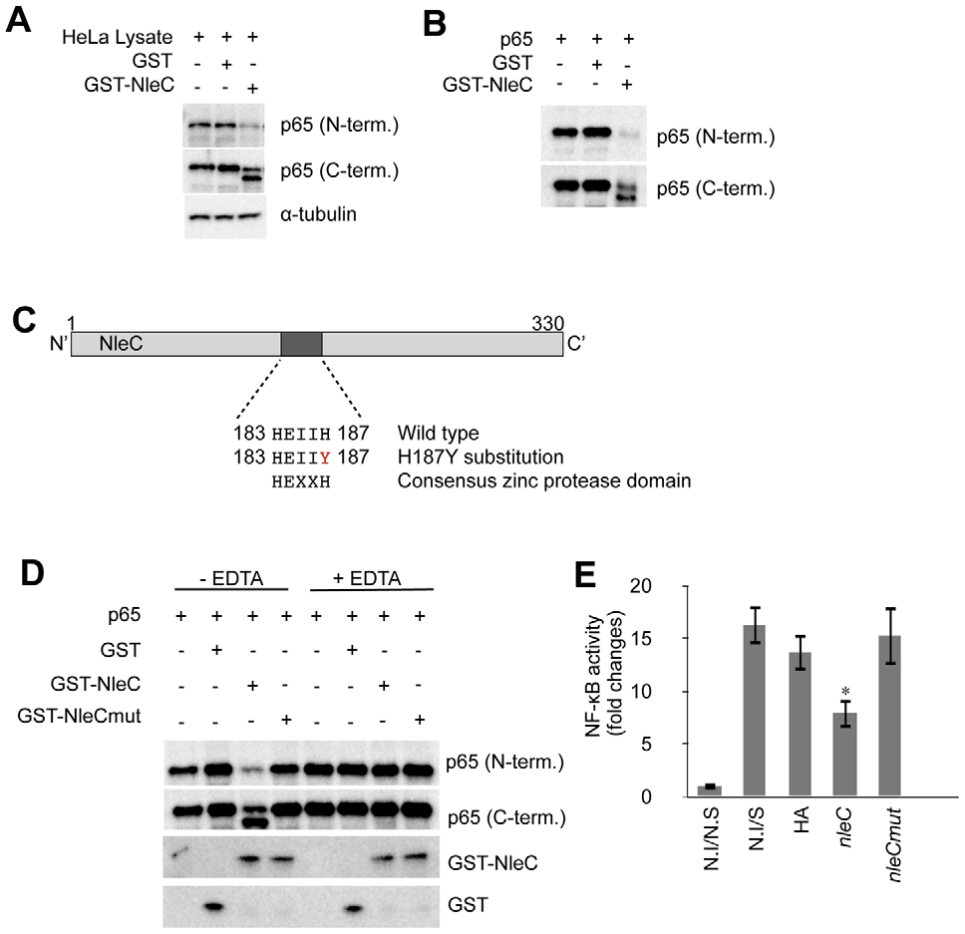
NleC cleaves p65 by its zinc protease domain. (A) Activity of purified GST-NleC protein. For lysates, 1×10^6^ of unstimulated HeLa cells were lysed by NET150 (10 mM Tris-Cl pH 8.0, 0.5% Triton-X100, 150 mM NaCl, 10% glycerol) without EDTA or protease inhibitors. GST protein or GST-NleC protein expressed in bacteria was purified and mixed with the HeLa lysates. The reaction mixtures were incubated at 25°C for 8 hours and then analyzed with antibodies against p65 (N-terminal or C-terminal specific) and α-tubulin (as a loading control). (B) Cleavage of p65 by NleC alone. p65, GST, and GST-NleC were expressed in bacteria and purified. The p65 was then mixed with either GST or GST-NleC in the reaction buffer. The mixtures were incubated at 25°C for 8 hours and analyzed with anti-p65 (N-term.) and anti-p65 (C-term.) antibodies. (C) Zinc protease motif in NleC. Schematic diagram showing the zinc protease domain in NleC. A Histidine (H) at 187 was replaced with Tyrosine (Y) by site-directed mutagenesis. (D) NleC's cleavage activity required the zinc protease motif and divalent metal ions. p65, GST, GST-NleC wild type (GST-NleC), and GST-NleC mutant (GST-NleCmut) were expressed in bacteria and purified. The p65 was mixed with GST, GST-NleC, or GST-NleCmut in the reaction buffer. The cleavage reaction was performed at 25°C for 8 hours. Samples were analyzed with anti-GST and anti-p65 antibodies against either the N- or C-terminal region. For the EDTA inhibition, EDTA was included in the reaction buffer at a final concentration of 10 mM. (E) NF-κB activity in cells infected with the *nleC*mut-expressing strain. Two days prior to infection, HeLa cells were transfected with NF-κB and luciferase reporter plasmids. Forty-eight hours post-transfection, cells were infected with TOB02/HA, TOB02/*nleC*-HA (*nleC*), or TOB02/*nleC*mut-HA (*nleC*mut) for 3 hours. Cells were rinsed and incubated in fresh DMEM containing 0.1 mg/ml gentamicin and HKE (10^8^/ml). Cells were stimulated for 8 hours, then the medium and cell lysates were analyzed for NF-κB SEAP and Luciferase reporter activities. Non-infected/non-stimulated (N.I/N.S) and non-infected/stimulated (N.S/S) HeLa cells served as negative and positive controls, respectively.

To elucidate the relationship between NleC and p65-C, we carried out *in vitro* cleavage assays to recapitulate the observation made in TOB02/*nleC*-HA-infected HeLa cells. We constructed and purified a fusion protein of NleC with GST attached to its N-terminal end (GST-NleC). To confirm the activity of the purified protein, cell lysates prepared from unstimulated HeLa cells were incubated with the purified GST-NleC. Using N- and C-terminal-specific antibodies to p65, we detected a decrease in p65 and the appearance of p65-C only in the mixture containing GST-NleC (Fig. 4A).

Next, to determine the dependency of this cleavage on host co-factors, purified p65 was mixed with GST-NleC. While both the p65 only and GST-added control samples showed no change in total p65 nor the detection of p65-C, the GST-NleC-containing mixture had less p65, and p65-C was readily detected (Fig. 4B). Based on these observations, we concluded that NleC could mediate the direct digestion of p65.

We next performed a bioinformatic search for any known domains in NleC. The search predicted a zinc protease domain located between amino acids 183 and 187. This amino acid sequence, HEIIH, corresponds to the consensus sequence HExxH, where x is any amino acid. To test if this domain was responsible for mediating the cleavage of p65, we performed site-directed mutagenesis in which we replaced the second histidine with tyrosine (H187Y) (Fig. 4C). Since both histidines of HExxH come in contact with zinc ions, the mutation of either residue should disrupt the domain and render it non-functional [34]. We mixed purified p65 with GST, GST-NleC wild type (GST-NleC), or GST-NleC mutant (GST-NleCmut) *in vitro*. As shown in Figure 4D, although GST-NleC produced p65-C, GST-NleCmut did not. Moreover, 10 mM EDTA greatly impeded the function of GST-NleC, suggesting that divalent metal cations are necessary for its activity (Fig. 4D).

Finally, we used the NF-κB reporter to assay the necessity of the zinc protease domain and the protease activity of NleC in the NF-κB suppression. HeLa cells were infected with TOB02/HA, TOB02/*nleC*-HA, or TOB02/*nleCmut*-HA for 3 hours, stimulated, and then reporter activities measured. As shown in Figure 4E, whereas TOB02/*nleC*-HA significantly suppressed the NF-κB activation, TOB02/*nleCmut*-HA failed to do so, indicating that the mutant had lost the anti-inflammatory effect of intact NleC. Taken together, these results showed that the HEIIH region of NleC is a zinc protease domain necessary for cleaving p65 and suppressing the host NF-κB activity.

### NleC functions in wild-type EPEC- and EHEC-infected cells

**Figure 5:**
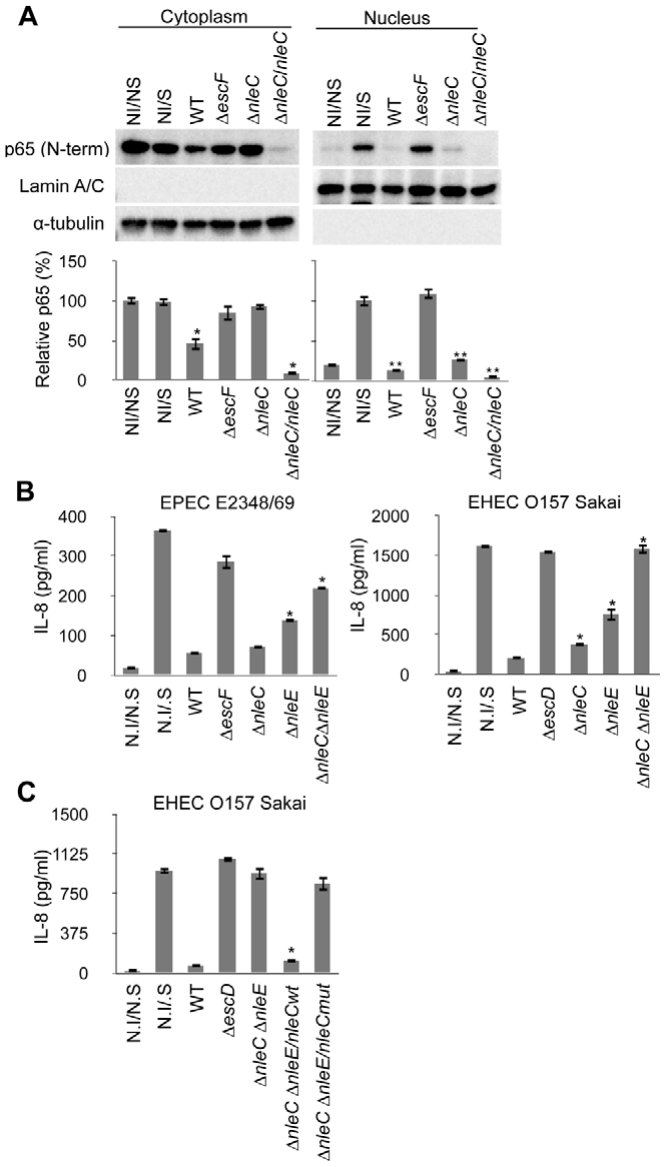
Concurrent deficiency in *nleE* and *nleC* impedes the immune-suppressive ability of EPEC/EHEC. (A) Amount of cytoplasmic and nuclear p65 protein in cells infected with *nleC* mutants. HeLa cells were infected with EPEC wild type (WT), Δ*escF* mutant, Δ*nleC* mutant, and Δ*nleC*/*nleC* complemented strains for 2 hours. At the end of the infection, TNF-α (50 ng/ml) was added, and the cells were further cultured for 40 min before being collected. The cytoplasmic and nuclear fractions were analyzed by anti-p65 (C-term.), anti-Lamin A/C and α-tubulin antibodies. This experiment was repeated three times and one of the representative blot is shown. For quantification, the total cytoplasmic p65 and the total nuclear p65 were normalized to the α-tubulin and Lamin A/C using ImageJ software. NI/NS (Non-infected/Non-stimulated) and NI/S (Non-infected/Stimulated) served as the negative and positive controls for p65 translocation in response to the TNF-α stimulation (* *p*<0.05, compared to cytoplasmic NI/NS; ** *p*<0.05, compared to nuclear NI/S). (B) IL-8 secretion by cells infected with EPEC or EHEC mutants. HeLa cells were infected with wild type, ΔT3SS mutant (Δ*escF* for EPEC; Δ*escD* for EHEC), Δ*nleC*, Δ*nleE*, and Δ*nleC* Δ*nleE* of EPEC and EHEC. Infections were proceeded for 3 hours for EPEC and 4 hours for EHEC strains. Cells were then rinsed and incubated in fresh DMEM containing 0.1 mg/ml gentamicin and HKE. Culture medium were collected 8 hours of stimulation and amount of IL-8 analyzed by ELISA. Non-infected/non-stimulated (N.I/N.S) and non-infected/stimulated (N.I/S) cells served as negative and positive controls (* *p*<0.05, compared to WT). Values of Δ*nleC* Δ*nleE* double KO mutant are not significantly different from ΔT3SS (*p*>0.05). (C) IL-8 secretion by cells infected with EHEC compound mutants. HeLa cells were infected with wild-type EHEC (WT), ΔT3SS mutant (Δ*escD*), Δ*nleC* Δ*nleE* mutant, or Δ*nleC* Δ*nleE* mutant harboring *nleCwt* or *nleCmut* plasmid for 4 hours. The cells were then rinsed and incubated in fresh DMEM containing 0.1 mg/ml gentamicin and HKE. After 8 hours of HKE stimulation, the culture supernatants were collected and analyzed for IL-8 by ELISA. Non-infected/non-stimulated (N.I/N.S) and non-infected/stimulated (N.I/S) cells served as negative and positive controls. (* p<0.05, compared to Δ*nleC* Δ*nleE*).

Using the reconstituted TOB02/*nleC*-HA strain, we discovered that NleC functions in host cells as a p65-targeting enzyme. Since the expression of *nleC* in the TOB02 strain is activated through an IPTG-inducible promoter, we wanted to know if we could observe a similar enzymatic activity when NleC was at its physio-pathologically relevant level, in infections with native strains of EPEC or EHEC. Therefore, we infected HeLa cells with wild type (WT), *ΔnleC*, or *ΔnleC*/*nleC*-HA EPEC for 2 h, and analyzed the amount of cytoplasmic and nuclear p65 in the cells using an anti-p65 (N-terminus) antibody after treatment of cells with HKE. We observed a reduction in cytoplasmic p65 in the WT-infected cells compared to cells infected with the *ΔnleC* mutant strain (Fig. 5A; left panel). As for nuclear p65, the amount of p65 was significantly reduced in both wild type and *ΔnleC* mutant infected cells compared to that of *ΔescF* (Fig. 5A; right panel). Next, we also analyzed the appearance of p65-C fragment in wild type-infected cells. When p65 in total lysate was detected using anti-p65 (C-term), we identified the generation of p65-C fragment in wild type infected cells, albeit in a lesser amount of that by *ΔnleC*/*nleC*-HA strains (Fig. S7). Taken together, we showed that NleC also functions as a protease targeting p65 in native strains. These results suggested that, even when NIeC was at physio-pathological levels, it could cleave p65, although the effect was less profound than that of the *nleC* complemented strain, presumably due to the difference in the amount of NleC present in the host cells.

### The EPEC Δ*nle*C Δ*nleE* compound mutant of EPEC and EHEC caused little reduction of NF-κB activation

After determining that NleC was an anti-inflammatory effector and identifying its functional zinc protease domain, we next examined whether NleC contributes to the immune suppression by EPEC/EHEC. For this, we generated an equivalent set of mutant strains in EPEC and EHEC, and measured the IL-8 secretion of cells infected with the EPEC/EHEC mutant. As shown in Figure 5B, the *ΔnleC* mutant of EPEC showed a comparable suppressor activity to the wild type EPEC. On the other hand, we observed statistically significant elevation of IL-8 secretion from the *ΔnleC* mutant of EHEC-infected cells compared to the WT EHEC-infected ones. We also infected HeLa cells with the *ΔnleE* mutant of EPEC and EHEC and observed increased IL-8 secretion, which is in agreement with the reported results of EPEC∶ *nleE* by Nadler et al and Newton et al [25], [26].

**Figure 6:**
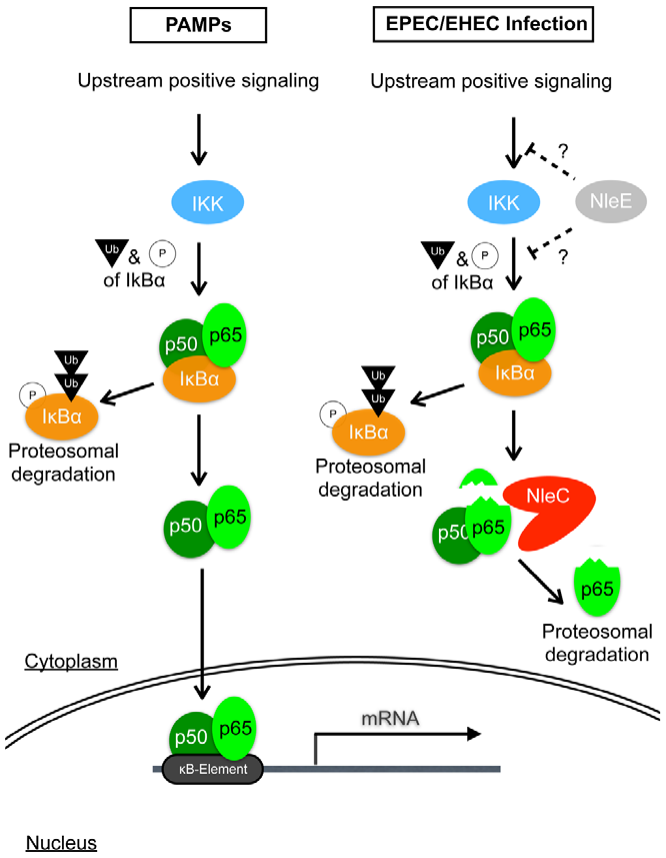
Schematic diagram illustrating the modes of action of NleC and NleE in host NF-κB suppression. Detection of PAMPs by cell surface receptors triggers the onset of the NF-κB signaling cascade, leading to a chain of events involving protein modifications and degradations; ultimately, activated p65/p50 NF-κBs enter the nucleus to transcribe various inflammatory genes, including IL-8. EPEC/EHEC utilizes two distinct T3SS-dependent non-LEE effectors to subdue this response. This is accomplished by the coordinated targeting of upstream and downstream components of the NF-κB pathway by NleE and NleC, respectively. NleE, by an unknown mechanism, retards the activation of IKK, thus retaining p65/p50 in a complex with IκB. NleC digests activated p65 and to a lesser extent the IκB-bound p65, to decrease the amount of NF-κB available to enter the nucleus, hence reducing the overall host response to the infection.

Although the IL-8 response by the host cells after the *ΔnleC* or *ΔnleE* mutant challenges was better, the inflammatory response was still only partial compared to that permitted by the *ΔescF* or *ΔescD* mutant. Based on the proposed actions of NleE and NleC as negative regulators of IKK activation and homeostasis of p65, respectively, and the observation that full host NF-κB activity was preserved during infection with TOE-A4, we predicted that NleE and NleC contributed the major portion of the suppression of pathogen-induced activation of NF-κB pathway. We therefore analyzed the total IL-8 secreted from HeLa cells infected with the *ΔnleC ΔnleE* (double knock-out) mutant. As shown in Figure 5B and 5C, whereas the wild type substantially inhibited the IL-8 secretion in infected cells, the infection of isogenic *ΔnleC ΔnleE* compound mutant resulted in a significantly diminished inhibition of IL-8 secretion compared to WT. Furthermore, the level of the IL-8 response exhibited by host cells infected with the compound mutant was comparable to that of cells infected by the T3SS defective mutant (*ΔescF* of EPEC and *ΔescD* of EHEC). The introduction of the *nleCwt* but not *nleCmut* expression plasmid into the *ΔnleC ΔnleE* mutant effectively restored the inhibition of IL-8 secretion by the infected cells. This major de-repression of the host NF-κB activation in the *ΔnleC ΔnleE* mutant suggested that these two T3SS-dependent, non-LEE effector proteins function in concert for the suppression of the NF-κB-signaling cascade and that the zinc protease domain is necessary for the anti-inflammatory function of NleC (Fig. 6).

## Discussion

Bacterial antigens of infecting pathogens can trigger intense local inflammation and the mobilization of immune cells. To prevent their early elimination, the EPEC and EHEC A/E pathogens control the host response by delivering anti-inflammatory bacterial proteins into the host cells. Although the recent identifications of NleH1 NleE and NleB showed that EPEC/EHEC use different mechanisms to modify the host immune response [24], [25], [26], those mechanisms did not completely account for the full immune-suppressive potential of these pathogens. Here, we found that NleC of EHEC and EPEC negatively modulates the host inflammatory response by reducing the amount of the mammalian p65 subunit of NF-κB. Specifically, this reduction is due to the direct cleavage of p65 by NleC, followed by proteasomal degradation of the cleaved p65. We also demonstrated that two type III secretion effectors, NleC and NleE, are necessary for and function in cooperation to achieve most of the immune suppression caused by these pathogens. By combining these effectors, EPEC and EHEC efficiently inhibit the activation of the NF-κB signaling pathway in infected host cells, thereby weakening the inflammatory response.

With the aim of discovering putative anti-inflammatory T3SS-dependent, non-LEE-encoded effector proteins, we employed both gain-of-function and loss-of-function approaches. In the gain-of-function analysis, we used a reconstituted bacterial system to study the effect of individual effector proteins on the host innate immune response. This method is preferable to transfection, because these artificial A/E bacteria bearing selected non-LEE effector genes faithfully recapitulated the infection and colonization of host cells in a T3SS-dependent manner. Moreover, because they expressed BFP, the reconstituted strain established colonies as quickly and efficiently as wild-type EPEC. As a result, the pathological action of the effectors could be compared over a similar time course with that of the native A/E pathogens. Another reason we avoided using transfection is that the expression levels may vary from cell to cell, and the transfection efficiency is difficult to maintain at a high level for all candidate genes, leading to possible false read-outs. Finally, the reconstitution method is also appropriate for isolating effectors with redundant functions. However, since amount and stability of translocated effector can not be controlled to be the same level, we can not rule out the presence of other effector(s) involving in modulation of inflammatory response by only this screening. Nevertheless, the use of this reconstituted system revealed not only NleC as a novel anti-inflammatory factor, but also the already-reported NleE [25], [26].

In the loss-of-function analysis, we used a set of EPEC mutant constructs with serial deletions of the pathogenic islands. This approach allowed the identification of multiple cooperative effectors that may have similar functions. Using this series of deletion mutants, we identified the EPEC mutant TOE-A4, which lacked four horizontally transferred elements that included nine non-LEE effector genes, and was as incapable of inhibiting NF-κB activation as the T3SS-deficient mutant. This complete loss of immune suppressing activity was also seen when NleE-NleB were deleted from TOE-A3. In contrast, Nadler et al. showed only a partial alleviation of the suppressed host inflammatory response using an EPEC Δ*nleE* Δ*nleB* double mutant [25]. The inconsistency between our findings in TOE-A4 and theirs may lie with the genes that had already been deleted from TOE-A3. Because we ruled out *espJ*, *nleF*, *nleG*, *nleH1*, *nleD*, *espL*, and *nleB*, based on our primary screening using the TOB02 strains, we speculate that the effectors responsible for most of the immune-suppressive effect exerted by EPEC or EHEC in response to HKE are NleC and NleE. Indeed, we showed that compound EPEC and EHEC mutants lacking both NleC and NleE (Δ*nleC* Δ*nleE*) were incapable of suppressing the host IL-8 secretion to a similar degree as the ΔT3SS (Δ*escF*) mutant after being stimulated with HKE, which engaged and activated mainly TLR-/IL-1β -NF-κB signaling pathways. The introduction into this compound mutant of either an *nleC*- or *nleE*- expressing plasmid restored the mutant's ability to suppress NF-κB activation. On the other hand, NleH1, another reported anti-inflammatory effector was not identified in these screenings. For verification, we generated an EHEC mutant deficient in NleH1 (Δ*nleH1*) and tested its ability to suppress the host IL-8 secretion. We did not detect any apparent difference in effect between the wild type and Δ*nleH1* (see Fig. S8). Therefore, the use of the EPEC mutant series not only helped us narrow down the list of candidate effectors substantially, but also, in conjugation with the TOB02 system, allowed us to identify NleC as a novel anti-inflammatory non-LEE effector.

Since IL-8 production is dependent on NF-κB activation, we examined the NF-κB signaling pathway to understand the mechanism of the NleC-dependent anti-inflammatory effect. We found that cells infected with NleC-expressing bacteria had markedly reduced active (phosphorylated) p65 as well as total p65, and that this reduction was due to the cleavage of p65 by NleC and the degradation of the remaining p65 fragments by proteasome. Thus far, caspase-3 is the only reported host factor that can cleave p65 similarly at the N-terminal region [33]. However, we showed that this cleavage event was not due to caspase-3, because the reduction of p65 was not prevented when the cells were pre-treated with a pan-caspase inhibitor. On the other hand, our bioinformatic search for known domains in NleC suggested the presence of a region (183HEIIH187) corresponding to a known zinc protease domain. By *in vitro* cleavage assays, we showed that this zinc protease domain of NleC is crucial for the processing of p65 and that the addition of a metal ion chelating agent, such as EDTA, abrogated the wild-type NleC function. Furthermore, it appears that the protease activity of NleC is selective as it could not digest p50, another common NF-κB subunit nor IκB (see Fig. S10). This inability to digest IκB suggests that the reduced IκB in cells infected with NleC-expressing bacteria may be of indirect consequences. These results clearly indicated that NleC is a metalloproteinase with an HEIIH domain that is necessary for its activity. Furthermore, the H187Y mutant of NleC lost its cleavage activity and the ability to suppress NF-κB activation. Therefore, we conclude that the p65 inhibitory function of NleC relies on this zinc protease domain.

As the NF-κB signaling pathway consists of multiple components, pathogens have evolved multiple strategies to subvert this system. For example, OspG and OspF of *Shigella* spp. respectively inhibit the ubiquitination of IκBα and modify the epigenetic information on the promoters of NF-κB-associated transcriptions [35], [36]. In Chlamydia, CT441 is a T3SS-secreted protease that can cleave p65 at its C-terminal region, generating p40 and p22 fragments [37]. Whereas p22 is degraded by proteasomes, p40 appears stable and inhibits the NF-κB activity when over-expressed [37]. Although CT441 and NleC share the same host target, these two effectors exhibit completely different pathological kinetics, possibly owing to differences in protease structures and their target sequences. We are currently attempting to elucidate the site of cleavage by NleC. Whereas NleC functions relatively early during bacterial colonization, CT441 acts at the mid-to-late stage of Chlamydia infection. However, since it is degraded by proteasomes upon being generated, it is unlikely that p65-C could exist long enough in the cell to inhibit NF-κB activity. Thus, NleC seems to be a novel type of virulence factor/effector which is evolved independently on these other NF-kB-targeting factors.

In our study, we showed that EHEC *ΔnleC* but not EPEC *ΔnleC* has statistically significant relief on host IL-8 response; this discrepancy between these two close-related pathogen may due to differences in their infection efficiency and amount of other translocated anti-inflammatory effectors, such as NleE or NleB. Nevertheless, the partial preservation of IL-8 secretion in cells infected with either the Δ*nleC* or Δ*nleE* mutant compared to its full preservation in cells infected with a ΔT3SS mutant shows that multiple bacterial effector proteins are needed for full inflammatory suppression. Based on our current understanding that NleE and NleC target different components of the NF-κB pathway, we believed that the contributions of NleE and NleC to the immune suppressiveness of EHEC/EPEC are additive. Indeed, when EPEC/EHEC mutants lacking both NleC and NleE (Δ*nleC* Δ*nleE*) were tested in the infection assay, the cells responded with a level of IL-8 secretion that was as high as in cells infected with a ΔT3SS (Δ*escF* or Δ*escD*) mutant. This near-total loss of inflammation suppression was also seen with EPEC TOE-A4, which is deficient in both *nleC* and *nleE* genes. These results demonstrated that EPEC/EHEC utilizes mainly these two effectors to suppress NF-κB activation triggered by TLR-/IL-1β pathways.

The mode of actions of NleE and NleC in NF-κB interference is becoming clear. NleE was shown to stabilize IκB and retain p65 in the IκB-p65 complex even after stimulation with TNF-α [25], [26]; however, the precise role of NleE as a direct or indirect regulator remains to be determined. On the other hand, NleC cleaves the N-terminus of p65, and thereby triggers p65's proteasomal degradation. Not only can NleC digest free p65, but when NleC is present in large amounts, it can also digest IκB-bound p65 (see Fig. S9), strongly suggesting that NleC can function whether p65 is in an inhibited or free state. However, considering NleE's ability to cause the retention of IκB (i.e. increasing the IκB-bound p65), we speculate that NleC cleaves released p65 more efficiently than the p65 bound by IκB. This is supported by our finding that little p65-C was observed after inhibiting the proteasome activity of a wild-type-infected cells. Since the expression of NleC in the reconstituted or complemented TOE-A4 strains showed a similar competency to reduce NF-κB activity as the strain expressing NleE, NleC is a potentially efficient inhibitor of NF-κB activation. Thus, in the wild-type pathogen, NleC, functioning downstream of NleE, may act by preferentially targeting and decreasing the number of active p65 molecules released by activated IKK that escapes the NleE inhibition (Fig. 6).

In our study, we did not identify NleB. As proposed by Nadler et al., NleB functions as an accessory factor to enhance inflammatory suppression of NleE in infected cells that were challenged by TNF-α [25]; and Newton *et al.* further demonstrated that NleB could also act independently to suppress TNF-α but not IL-1β induced activation of NF-κB [26]. Based on their studies, it has been suggested that NleE works either directly or indirectly targeting the activation of IKK complex and IκB degradation and that NleB interferes the upstream components of TNF-α signaling pathway. The likely explanation for our inability to identify NleB may due to the use of different stimulant than theirs, i.e. the heat-killed bacteria, to provoke the second inflammatory response following bacterial infection of HeLa cells. As HKE contain bacterial compounds, such as LPS, flagellin, unmethylated bacterial DNA, and RNA, these mainly trigger the activation of NF-κB via TLR-/IL-1β associated pathways. Therefore, the use of TNF-α in our screenings will likely yield similar results as presented by Nadler et. al and Newton et al. [25], [26]. Nevertheless, as epithelial cells first come in contact with antigens of bacterial components at the onset of infection, the anti-inflammatory NleE and NleC at the level of IKK/IκB/NF-κB cascade are important for executing successful infection by EPEC/EHEC.

Several studies have examined the *in vivo* role of *nleC* using animal models and found no clear evidence showing the necessity of NleC in the bacterial colonization [38], [39]. As shown in our *in vitro* experiments, deletion of *nleC* gene alone shows only little or no difference in suppression of inflammatory response. This explains the reason of no apparent effect of *nleC* deletion in colonization in animal models. Although we are yet to provide *in vivo* evidence of *ΔnleC ΔnleE* double mutant, it is speculated that this mutant would be highly attenuated and causes early elimination by the host due to the increase of recruited immune cells to the sites of infection.

In conclusion, we showed that NleC negatively modulates the host NF-κB activity by the direct enzymatic digestion of p65. We also demonstrated that NleE and NleC function in concert to interfere with the NF-κB pathway, and that these two molecules are responsible for anti-inflammatory effect of EPEC/EHEC. The presence of alternative factors for modulating the NF-κB activation pathway in EPEC/EHEC indicates that the manipulation of cell signaling must be important for successful infection. The outcome of the inflammatory response to infection depends on multiple factors contributed by both the host and the pathogen. Our study showing how NleE and NleC interfere with the host innate immune response not only illustrates the importance of the NF-κB pathway, which functions at the center of the confrontation, but also broadens our understanding of the intricate interplay between pathogen and host.

## Materials and Methods

### Bacterial strains, plasmids, cell culture, and oligonucleotides

The bacterial strains and plasmids used in this study are described in Table S1. Primers used for cloning are described in Table S2. DNA fragments containing *nleC* (ECs0847), *nleE* (ECs3858), *espL2* (ECs3855), and *nleB1* (ECs3857) were amplified directly by PCR from EHEC Sakai chromosomal DNA (Accession No. NC_002695). The PCR products were subcloned into the pHA-CTC plasmid (Table S1), resulting in pHA-NleC, pHA-NleE, pHA-EspL, and pHA-NleB. The NleC (ECs0847) PCR fragment was also subcloned into pGEM-T (Promega), pEGFP-C1 (Clontech), and pGEX-6P (GE Healthcare) to yield pGEM-T-NleC, pEGFP-NleC, and pGEX-6P-NleC. For the site-directed mutagenesis generating H187Y in NleC, pGEM-T-NleC was used as the template and amplified with primers designed to change the second histidine to tyrosine, yielding pGEM-T-NleCmut. The NleCmut fragment was excised and cloned into pGEX-6P to obtain pGEX-6P-NleCmut. The same fragment was also subcloned into pHA-CTC to generate pHA-NleCmut. To construct pET21A-RelA, RelA was excised from pEv3s-T7-RelA (obtained through Addgene, plasmid # 21984) and cloned into the pET21A plasmid. The DNA sequences were verified by sequencing. The primers used are listed in Table S2. The human cervical cancer cell line HeLa was maintained in MEM (Sigma) supplemented with 10% FCS (Sigma) and 0.1 mM non-essential amino acids (Invitrogen). Request for EPEC strains with a series of deletion and recombinant *E. coli* K12 strain harboring LEE and bfp (TOB01 and TOB02) should be sent to Tetsuya Hayashi of University of Miyazaki.

### Construction of mutants derived from strain E2348/69

We constructed a series of mutants from EPEC strain E2348/69 (Table S1) as described previously by Sekiya *et al.* (2001) [40] with some modifications. For example, to construct an *nleC* deletion mutant (strain TOE-S1), we amplified upstream and downstream regions of the *nleC* genes (about 700-bp each and including a short sequence encoding the N- or C-terminal part of the protein) by PCR using the nleC_B1F (5′-aaaaagcaggctTCTATCGGGAAGATGTTGA-3′)/nleC_B1R (5′-TGCAAAGACGAATCATCGCATGTTTATATCTAATACCCT-3′) and the nleC_B2F (5′-CGATGATTCGTCTTTGCA-3′)/nleC_B2R (5′-agaaagctgggtGATTCAATAGCATTCAGGAG-3′) primer pairs, respectively. As the nleC_B1R primer contained an 18-base sequence complementary to the nleC_B2F primer sequence (underlined), the resulting two PCR products shared an identical 18-base sequence at their right and left ends. By the joint-PCR method [41] using the two PCR products as a template and the adapt-F (5′-GGGGACAAGTTTGTACAaaaaagcaggct-3′)/adapt-R (5′-GGGGACCACTTTGTACAagaaagctgggt-3′) primer pair, we obtained a chimeric PCR product (referred to as Δ*nleC* fragment) consisting of the upstream and downstream sequences of the *nleC* gene. At this stage, an in-frame deletion was introduced into the target gene. Using the adaptor sequences in the adapt-F and -R primers (indicated by lower-case letters), we cloned the Δ*nleC* fragment into the pDONR201 entry vector by BP clonase II (Gateway cloning system: Invitrogen). The Δ*nleC* fragment-containing pDONR201 derivative and the Not1/NcoI double digested pABB-CRS2 vector were incubated with LR clonase II (Invitrogen) to transfer the Δ*nleC* fragment from pDONR201 to pABB-CRS2 (a R6K-derived positive suicide vector). The Δ*nleC* fragment-containing pABB-CRS2 derivative was introduced into *E. coli* SM10λ*pir* and then transferred to strain E2348/69 by conjugation. The transductants were first screened on M9 minimum plates containing 0.8% glucose (host marker) and ampicillin (Ap; 50 µg/ml) to obtain clones in which the pABB-CRS2 derivative was integrated into the targeted chromosomal region of E2348/69. These clones were grown on LB plates containing 5% sucrose to obtain clones in which the pABB-CRS2 plasmid was eliminated by homologous recombination. Finally, among the Ap-sensitive and sucrose-resistant clones, we screened for clones that contained an in-frame deletion in the *nleC* gene by PCR and sequencing analysis using the nleC_ckF/nleC_ckR primers. Other mutants were also constructed from E2348/69 or its derivatives by the same method using the primers listed in Table S2. Since multiple T3SS effector genes are present in a cluster on many of the prophages (PPs) and integrative elements (IEs) in E2348/69, we deleted these gene clusters en bloc (strains TOEA1 to TOEA7).

### Construction of strains TOB01 and TOB02

A fosmid library of EPEC strain B171-8 was constructed using the CopyControl Fosmid Library Production Kit (Epicentre, Madison, WI), as described previously [10]. Fosmid clones containing part or all of the LEE element were screened by PCR using the *eae* gene-specific primers (SK1 and SK2 in Table S2). The end sequences of all the *eae*-positive clones were determined using the FosF and FosR primers (Table S2), to select a clone containing the entire LEE element of B171-8 (referred to as pTOK-02). We then introduced a plasmid named pTOK-01, which contained the *bfp* operon and the *perA*-*C* locus, encoding the genes for bundle forming pilus biosynthesis and a positive regulator (*perC*) for LEE gene expression, respectively, into an EPI300-T1-derivative containing pTOK-02, to obtain strain TOB02. To construct plasmid pTOK-01, we purified the pB171 plasmid from B171-8 using the Qiagen Plasmid Midi kit (Qiagen, Tokyo, Japan) and digested it with SmaI. A 22-kb fragment containing the *bfp* operon and the *perA-C* locus was separated by Pulsed-field gel electrophoresis (PFGE), extracted from the PFGE gel using beta-agarase I (Nippon Gene Co., LTD, Tokyo, Japan), and cloned into the EcoRV site of pWKS130 [42] using the BKL kit (Takara Bio Inc., Shiga, Japan). The recombinant plasmid (pTOK-01) was introduced into *E. coli* strain DH10B (Electrocomp GeneHogs *E. coli* DH10B; Invitrogen) by electroporation. Finally, the pTOK-01 plasmid was purified using the QIAprep Spin Miniprep kit (Qiagen) and used to construct strain TOB02, described above. Strain TOB01 was constructed by introducing a pCC1FOS fosmid vector (constructed by self-ligation) and the pTOK-01 plasmid into *E. coli* strain EPI300-T1 by electroporation. More details of the genetic structures of the inserted fragments in the pTOK-01 and pTOK-02 plasmids are shown in Figure S1.

### Secretion assay

TOB02/HA and TOB02/nleC-HA were cultured in Luria-Bertani broth overnight with constant agitation at 37°C. Bacterial cultures at stationary phase were diluted 100-fold in serum-free DMEM (Sigma) and cultured with 120× rpm agitation at 37°C until the O.D_600_ reached 1.0. The bacterial cultures were separated into the cell pellet (whole cell) and the culture supernatant (supernatant) fractions by centrifugation at 8,000 rpm. The supernatant was filtered (pore size, 0.22 µM) and concentrated by the addition of trichloroacetic acid (Sigma) and deoxycholic acid (Wako) to a final concentration of 6% and 0.05%, respectively. The precipitates were re-dissolved in acetone (Wako) and centrifuged. Finally, after removing the acetone, the residual precipitates were dissolved in 2× SDS sampler buffer. The whole cells were also lysed with 2× SDS sampler buffer (100 µl per O.D_600_ unit of original culture).

### IL-8 ELISA

HeLa cells were seeded at a density of 2×10^5^ cells/well in 24-well plates. The next day, overnight-grown bacteria were inoculated into serum-free DMEM and shaken for 2 hrs at 37°C (Pre-activation). During the pre-activation period, the cell medium was changed to serum-free DMEM, and the cells were cultured at 37°C, 5%CO_2_ until the beginning of infection. The cells were subjected to bacterial infection at moi (multiplicity of infection) of 100 for 3–4 hours. Infection was terminated by washing the cells with PBS to remove non-adherent bacteria, and the medium was replaced with fresh DMEM containing gentamicin (0.1 mg/ml) and HKE (heat-killed *E. coli*) at concentration of 10^8^ bacteria/ml. The cells were further cultured for 8 to 12 hours, and the medium was collected for the analysis of IL-8 by ELISA, performed according to the manufacturer's protocol (Thermo Scientific). All the experiments were performed in triplicate and repeated three times. Student's t-test was used to calculate the significance.

### SEAP reporter assay

HeLa cells were first seeded at 1×10^5^/well in 24-well plates one day prior to the transfection. The pNFKB-SEAP reporter plasmid (Clonetech; containing the κB-binding element and SEAP reporter gene) and pGL4-13 control luciferase plasmid (Promega) were transfected using Lipofectamine 2000 (invitrogen), according to the manufacturer's protocol. Forty-eight hours after transfection, the cells were infected as described above for the IL-8 ELISA, except that at the end of stimulation, the cell medium and lysates were collected and assayed for reporter activity. SEAP (for NF-κB activity) and Luciferase (transfection control) were analyzed using the Great EscApe SEAP Fluorescence Detection kit (Clontech) and ONE-Glo luciferase assay system (Promega), respectively. The NF-κB activities were normalized using the luciferase reporter. For the EGFP-*nleC* SEAP/Luciferase assay, pNFKB-SEAP and pGL4-13, pEGFP-C1, or pEGFP-NleC were co-transfected into HeLa cells. Forty-eight hours later, the cell medium was changed to serum-free DMEM for 2 hours. The cells were then stimulated with HKE for 7 hours. The SEAP and Luciferase activity were measured as described above. All experiments were performed in triplicate and repeated three times. The student's t-test was used to calculate the significance.

### In vitro cleavage assay

p65, GST, GST-NleC, and GST-NleCmut were expressed by bacteria. p65 was purified by Dynabeads (Invitrogen) conjugated to anti-p65 antibodies (ab7970, Abcam); GST, GST-NleC, and GST-NleCmut were purified by GST sepharose beads (GE Healthcare). For the *in vitro* cleavage assay, p65 (50 nM) was mixed with GST (0.3 nM), GST-NleC (0.3 nM), or GST-NleCmut (0.3 nM) in a final volume of 20 µl of reaction buffer (10 mM Tris-HCl pH 7.4, 150 mM NaCl, 0.5 mM DTT, 2.5 mM CaCl_2_, and 0.5 mM MgCl_2_, 0.5 nM ZnCl_2_). The reaction mixtures were incubated at 25°C for 8 hours. In some samples, EDTA was added to the reaction buffer at final concentration of 10 mM.

### Immunoblotting

Unless otherwise specified, the infected or transfected HeLa cells were washed twice with ice-cold PBS, then directly lysed with 2× SDS sampler buffer. The lysates were sonicated briefly to reduce the viscosity of the genomic DNA. For cell fractionation, PBS washed cells were pelleted, resuspended in Buffer A (10 mM HEPES pH 7.9, 1.5 mM MgCl_2_, 10 mM KCl, 0.5 mM DTT, 0.05% Triton X-100 and 1× protease inhibitor cocktail), and centrifuged. The supernatant was collected as the cytoplasmic fraction. Then the remaining pellet was washed twice with buffer A and re-suspended in Buffer B (300 mM NaCl, 15 mM HEPES pH 7.9, 1.5 mM MgCl_2_, 0.2 mM EDTA, 0.5 mM DTT, 26% glycerol (v/v)). Samples were sonicated and left on ice for 30 min before being centrifuged. The resulting supernatant containing the nuclear content was collected. For western blotting, a standard protocol was used. Anti-p65 N-term (# 4764, Cell Signaling Technology), anti-phospho-p65 (# 3033, Cell Signaling Technology), anti-IκBα (# 4814, Cell Signaling Technology), anti-Lamin A/C (# 2032, Cell Signaling Technology), anti-p65 C-term (ab7970, Abcam), and anti-α-tubulin (Clone B-5-1-2, Sigma) antibodies were used.

### Immunofluorescence staining

HeLa cells were seeded at density of 1×10^5^ cells/cm^2^ on cover glasses one day prior to the experiment. On the day of experiment, culture media were exchanged with serum free DMEM and cells were subjected to pre-activated bacteria at m.o.i of 100. Infection was allowed to proceed for 3 hours and was terminated by washing cells with PBS to remove non-adherent bacteria. Cells were fixed with 4% paraformaldehyde and permeabilized with 0.5% Triton X-100 in PBS. Following the blocking with 5% BSA in PBS, cells were stained with anti-HA (Bethyl Laboratories, Inc., Montgomery, TX, USA), Rhodamin-Phallodin (Wako, Japan), and DAPI (Sigma-Aldrich, Japan). Anti-rabbit-FITC (against anti-HA antibody) was used as the secondary antibody. Immunofluorescence images were taken using BioRad Radiance2100 confocal microscope.

## Supporting Information

Table S1 Bacterial strains and plasmids used in this study.
(0.05 MB PDF)

Table S2 Primers and oligonucleotides used in this study.
(0.02 MB PDF)

Figure S1 Gene organizations of the inserted fragments in pTOK-01 and pTOK-02.
(0.73 MB PDF)

Figure S2 Comparison of Tir translocation efficiency between wild type EPEC and reconstructed strain TOB02. HeLa cells were infected by m.o.i of 100 of pre-activated wild type EPEC, Δ*escF* (T3SS defective mutant), and TOB02 for 2 hours. Cells were rinsed with cold PBS twice to remove the non-adherent bacteria, scrapped and collected in the presence of sonication buffer (50 mM NaCl, 50 mM Tris pH 7.9, 10% sucrose (w/v), 2 mM EDTA, 0.4 mM Na_3_VO_4_, 1× protease inhibitor cocktail). Samples were sonicated for 3 sec to disrupt mammalian but not bacterial membrane. Then ultracentrifugation was applied at 45,000 rpm, 15 min. at 4°C. The pellet was washed once with sonication buffer and resuspended in the lysis buffer (50 mM NaCl, 50 mM Tris pH 7.9, 10% sucrose (w/v), 2 mM EDTA, 0.4 mM Na_3_VO_4_, 0.5% Triton-X, and 1× protease inhibitor cocktail). Lysates were subjected to second round of ultracentrifugation (45,000 rpm, 5 min. at 4°C). Finally the supernatant which contained the membrane fraction (host cell membrane) was collected. For the bacterial whole cell (bacteria), the bacterial pellet from 1 ml of the activation culture was lysed with 2× SDS sampler buffer. Samples were analyzed by immunoblotting using anti-Tir antibody. This experiment was repeated three times and the representative blot is shown.
(0.08 MB PDF)

Figure S3 TOB02/*nleC*-HA is able to secret NleC-HA via a reconstituted T3SS. Overnight-grown TOB02/HA and TOB02/*nleC*-HA were inoculated into DMEM for the secretion assay. When the bacterial growth reached O.D_600_ ∼1.0, the supernatant and bacterial pellet were separated by centrifugation and subjected to immunoblot analysis. An anti-HA antibody was used to detect the expression of NleC-HA, and anti-DnaK was used to verify that the supernatant was free of whole-bacteria contaminants.
(0.09 MB PDF)

Figure S4 NleC-HA can be secreted by the endogenous T3SS in EHEC O157:H7, and localizes to the cytoplasm. The NleC-HA-expressing wild-type EHEC O157:H7 was used to infect HeLa cells. After 4 hours of infection, the cells were washed with PBS to remove non-adherent bacteria, and fixed with 4% PFA (paraformaldehyde). The cells were then stained with anti-HA (FITC) and with DAPI to label DNA. Photographs and the xz-axis views were taken using a Radiance 2100 confocal laser scanning microscope (Bio-Rad).
(0.34 MB PDF)

Figure S5 The degradation of p65-C is dependent on the proteosome. HeLa cells were first pretreated with either DMSO, or MG132 (5 µM), or lactacystin (10 µM) for 2 hours. Pre-activated TOB02/HA or TOB02/nleC-HA was used to infect the cells for another 2 hours. The cells were rinsed with PBS to remove non-adherent bacteria and lysed directly by addition of SDS sampler buffer. Samples were analyzed using anti-p65 (C-term) and anti-alpha-tubulin antibodies.
(0.08 MB PDF)

Figure S6 Caspase-3 is not involved in p65 cleavage during the TOBA2/*nleC*-HA bacterial infection. HeLa cells were first pretreated with either DMSO or z-VAD-fmk (5 µM) for 2 hours. Pre-activated TOB02/HA or TOB02/*nleC*-HA was then used to infect the cells for another 2 hours. The cells were rinsed with PBS to remove non-adherent bacteria and lysed by the direct addition of SDS sampler buffer. The cell samples were analyzed using anti-p65 (C-term.) and anti-Lamin A/C antibodies by immunoblotting.
(0.10 MB PDF)

Figure S7 Generation of p65-C in native wild-type EPEC. HeLa cells pretreated with either DMSO or MG132 (5 µM) were infected with EPEC of wild type (WT), *ΔescF, ΔnleC*, and *ΔnleC/nleC* for 2 hours. Non-adherent bacteria were removed by washing cells with PBS. Cells were then directly lysed with sample buffer and analyzed by anti-p65 (N-term), anti-p65 (C-term), and α-tubulin (loading control) antibodies. Short and Long exposure of the membrane were taken. p65-C fragment is indicated by arrowheads. This experiment was repeated three times and one of the representative blot is shown.
(0.11 MB PDF)

Figure S8 EHEC Δ*nleH1* does not alleviate the immune suppression. HeLa cells were infected with pre-activated wild-type, or the Δ*escF*, Δ*nleH1*, and Δ*nleH2* isogenic mutants of EHEC. After 4 hours of infection, the cell culture medium was replaced with fresh DMEM containing gentamicin (0.1 mg/ml) and HKE (1/10 vol.). The cells were further cultured for 8 hours, and the medium was then collected for IL-8 analysis by ELISA. Similar experiments were performed in triplicate and repeated three times. Student's t-test was used, and p<0.05 was considered significant.
(0.04 MB PDF)

Figure S9 IκBα-associated p65 can be processed by NleC. HeLa cells pretreated with either DMSO or MG132 (5 µM) were infected with wild-type, Δ*nleC,* or Δ*nleC*/p*nleC*-HA of EPEC for 2 hours. At the end of infection, the HeLa cells were rinsed with PBS to remove non-adherent bacteria, and lysed with NET150 on ice. The cytoplasmic fraction was immunoprecipitated with an anti-IκBα antibody, and the pull-down products were analyzed using an anti-p65 (C-term.) antibody by immunoblotting.
(0.09 MB PDF)

Figure S10 NleC does not cleave p50 nor IκB *in vitro*. Cell lysates were prepared from unstimulated HeLa cells. The lysates were then mixed with GST, GST-NleCwt, and GST-NleCmut in vitro and were incubated at 25°C for 8 hours. The reactions were terminated by direct addition of 2× sample buffer. Samples were analyzed by immunoblotting using anti-p50, anti-IκBα, and anti-tubulin antibodies. This experiment was repeated three times and the representative blot is shown.
(0.07 MB PDF)
