# Supplementary material for: NleC, a Type III Secretion Protease, Compromises NF-κB Activation by Targeting p65/RelA
Source: PLoS Pathog. 2010 Dec 16;6(12):e1001231. doi: 10.1371/journal.ppat.1001231 (PMC3002990; doi:10.1371/journal.ppat.1001231)
Supplement: Table S1 — Bacterial strains and plasmids used in this study. (0.05 MB PDF) [file ppat.1001231.s001.pdf]

Table S1 Bacterial strains and plasmids

name	description	reference
EHEC strains		
RIMD0509952	EHEC wild type O157:H7 Sakai	Hayashi et al. (2001)
SKI1101	EHEC Δ*escF*	this study
SKI1756	EHEC Δ*nleC*	this study
SKI1757	EHEC Δ*nleE*	this study
SKI1758	EHEC Δ*nleC* Δ*nleE*	this study
EPECstrains		
E2348/69	EPEC wild type	Iguchi et al.(2009)
TB0661	E2348/69 Δ*escF*	this study
TOE-S1	E2348/69 Δ*nleC*	this study
TOE-S4	E2348/69 Δ*nleE*	this study
TOE-S2	E2348/69 Δ*nleC* Δ*nleE*	this study
TOE-A1	E2348/69-derivative: the *nleE*/*nleB*/*espL* cluster (on IE2)	this study
TOE-A2	derivative of TOE-A1: the *nleH*/*cif*/*espJ* cluster (on PP2)	this study
TOE-A3	derivative of TOE-A2: the *nleG*/*nleC*/*nleD* cluster (on PP4)	this study
TOE-A4	derivative of TOE-A3: the *nleE*/*nleB*/*espL* (on IE6) deleted	this study
TOE-A5	derivative of TOE-A4: the *nleH*/*nleA*(*espI*)/*nleF*/*espO* cluster	this study
TOE-A6	derivative of TOE-A5: the *espG2* (orf3) (on IE5) deleted	this study
TOE-A7	derivative of TOE-A6: the *espG* gene (on LEE) deleted	this study
B171-8	EPEC wild type	Puente et al. (1996)
TB0378	B171-B Δ*escC*	this study
E.coli K12 strains		
EPI300-T1	F– *mcrA* Δ(*mmr-hsdRMS-mcrBC*) 80d*lacZ*ΔM15 Δ*lacX74* *recA1 endA1 araD139* Δ(*ara,leu*)7697 *galU galK* λ– *rpsL* *nupG trfA tonA* d*hfr*	Epicentre
TOB01	EPI300-T1-derivative: contains pCC1FOS vector (self-ligated) and pTOK-01	this study
TOB02	EPI300-T1-derivative: contains pTOK-02 and pTOK-01	this study
Plasmids		
pHA-CTC	HA fusion plasmid: a derivative of pFLAG-CTC (Sigma)	this study
pHA-NleC	pHA-CTC derivative: NleC-HA expressing plasmid	this study
pHA-NleE	pHA-CTC derivative: NleE-HA expressing plasmid	this study
pHA-EspL	pHA-CTC derivative: EspL2-HA expressing plasmid	this study
pHA-NleB	pHA-CTC derivative: NleB1-HA expressing plasmid	this study
pWKS130	Low-copy-number cloning vector, km, pSC101ori	Wang and Kushner (1991)
pDONR201	Gateway donor vector, *ccdB*, Km	Invitrogen
pABB-CRS2	positive suicide vector, Ap, *sacB, R6Kori*	Sekiya *et al.*(2001)
pTOK-01	pWKS130-derivative: contains the 22 kb fragment (containing the *bfp* operon and *perA*, *B*, *C* genes) of Sma*I*-digested pB171 plasmid of B171-8	this study
pTOK-02	pCC1FOS-derivative: contains the LEE element of B171-8	this study

Request for strains of E2348/69 derivatives and TOB01, TOB02 should be sent to Tetsuya Hayashi, Miyazaki University.

Hayashi T, Makino K, Ohnishi M, Kurokawa K, Ishii K, et al. (2001) DNA Res 8(1): 11-22

Iguchi A, Thomson NR, Ogura Y, Saunders D, Ooka T, et al. (2009) J Bacteriol 191(1): 347-354

Puente JL, Bieber D, Ramer SW, Murray W, Schoolnik GK (1996) Mol Microbiol 20(1): 87-100

Wang R. F., and Kushner S. R. (1991) Gene 100: 195-199

Sekiya, K., Ohishi, M., Ogino, T., Tamano, K., Sasakawa, C., and Abe, A. (2001) Proc. Natl. Acad. Sci. USA. 98, 11638-11643
