# Supplementary material for: NleC, a Type III Secretion Protease, Compromises NF-κB Activation by Targeting p65/RelA
Source: PLoS Pathog. 2010 Dec 16;6(12):e1001231. doi: 10.1371/journal.ppat.1001231 (PMC3002990; doi:10.1371/journal.ppat.1001231)
Supplement: Table S2 — Primers and oligonucleotides used in this study. (0.02 MB PDF) [file ppat.1001231.s002.pdf]

Table S2 Primers and oligonucleotides used in this study

oligonucleotide	sequence
for cloning and expression plasmid construction	
0847-N5	CATGCATATGAAAATTCCCTCATTACAGTC
0847-C5	GTTACTCGAGCTGATTGTGTTTGTCCACATCC
nleC-N-BamH1-1	CTTGGGATCCATGAAAATTCCCTCATTACAGTC
nleC-C-BglII	CTTCAGATCTTTATTGCTGATTGTGTTTGTCCACATC
nleC-C-XhoI	CACACTCGAGTCATTGCTGATTGTGTTTGTCCAC
0847-HEXXY-F	GCAGGAAGGACTGATTCACGAGATTATTTATCATGTTACTGGATCTAGCG
0847-HEXXY-R	CGCTAGATCCAGTAACATGATAAATAATCTCGTGAATCAGTCCTTCCTGC
nleE-N-Nde1	GTTGCATATGATTAATCCTGTTACTAATACTCAGG
nleE-C-XhoI	TGGTCTCGAGCTCAATTTTAGAAAGTTTATTATTTATGT
ECs3855-N4	GAAAGGATCCAAACATATGCCAATAATAAACAAATCGG
ECs3855-C4	CCCGGCTCGAGATTGGAATAATAATTATATACATCGAG
ECs3857-N4	GAGAGAATTCACTGATATGAATTACTGAATGAGG
ECs3857-C4	GTGAGTCGACTGAACTGCAGGTATACATACTGGT
for EPEC deletion construction	
IE2_B1F	AAAAAGCAGGCTTCCTCGGCTTGAATAAGTTCC
IE2_B1R	TGTTCAATTTGAAACTGGCTGCAGCAACTTGTGTAAGGGACA
IE2_B2F	CAGCCAGTTTCAAATTGAACA
IE2_B2R	AGAAAGCTGGGTTCTTGACGCCCATGGAATAT
IE2_ckF	TGCATCAGCCCGAAGTTTTT
IE2_ckR	AATAACGGTACAGGGCATCA
IE5_B1F	AAAAAGCAGGCTCGAGCGGCTTTCTTCATAACT
IE5_B1R	TGTTATATTTCTGTCTGCCAGTTCGTCGCAACGAGTACATCT
IE5_B2F	CTGGCAGACAGAAATATAACA
IE5_B2R	AGAAAGCTGGGTTGCTGATTTCTGGAGCCATT
IE5_ckF	TTATTTGAGGCCGGCGAGAA
IE5_ckR	GTCATGCCTTTCGCAAACAA
IE6_B1F	AAAAAGCAGGCTAGCACAGGCCTCACACTCTT
IE6_B1R	ATTTTAGCCCCCCTACACAACCGCCTGACAGTGGAAATAAT
IE6_B2F	TTGTGTAGGGGGGCTAAAAT
IE6_B2R	AGAAAGCTGGGTTTCTGTACTCCAGCGTGAACG
IE6_ckF	ATGTAACAGTGCCAGGGGATA
IE6_ckR	ACATAGCTGACTTTGCCCGTT
PP2_B1F	AAAAAGCAGGCTTCTGACGGGAAATGCAGGT
PP2_B1R	TCATCCATATGAAGACAAGGACTCCGGTTTTGTGAAGCTGT
PP2_B2F	CCTTGTCTTCATATGGATGA
PP2_B2R	AGAAAGCTGGGTCGACTTTGGTAAAACCGGAT
PP2_ckF	TAATGCAGCCAGGGATGCAA
PP2_ckR	TCATCAGTAACGATCTGAGCG
PP4_B1F	AAAAAGCAGGCTACAGGGTCCAAAGGGAGATAC
PP4_B1R	AAGTTTTGTGTGCCTCCGTTCACCATGGCATGACAACAAA
PP4_B2F	AACGGAGGCACACAAAACTT
PP4_B2R	AGAAAGCTGGGTCCTGCCTCCAGCACTTTTAA
PP4_ckF	GAAGACGCCGTAAACCGAA
PP4_ckR	ACAGCACGTTCAGTGGTTTT
PP6_B1F	AAAAAGCAGGCTTATCTACCGTCAGTTGCGTCC
PP6_B1R	AAACTTTCCGCCCTAGCATTGCTGAAATGGATGACGGCTAT
PP6_B2F	AATGCTAGGGCGGAAAGTTT
PP6_B2R	AGAAAGCTGGGTTGTATCCGCACGCAGATTAA
PP6_ckF	CGACGCGTAATTATTATCTGG
PP6_ckR	TGTCAGAATAACGCCCTGCA
bfpA_B1F	AAAAAGCAGGCTACAAAAGGGCGCAGACCAT
bfpA_B1R	CCTCCCATATAATACGCCCAAAACCCCCGTATTAATAGGTCA
bfpA_B2F	TTGGGCGTATTATATGGGAGG
bfpA_B2R	AGAAAGCTGGGTACCTTCTAATTTACCGGGCA
bfpA_ckF	ACCCGCGTATACACCTCCAT
bfpA_ckR	TGAAGCGTTTAGTTTCCTCGT
nleC_B1F	AAAAAGCAGGCTtctatcgggaagatgttga
nleC_B1R	tgcaaagacgaaTCATCGCATgtttatatctaataccct
nleC_B2F	CGATGAttcgtctttgca
nleC_B2R	AGAAAGCTGGGTgattcaatagcattcaggag
nleC_ckF	agtcaaccaaaagactgct
nleC_ckR	ttgaggtgtattaccactg
nleE_IE6_B1F	AAAAAGCAGGCTtctaccgagcagatgtgg
nleE_IE6_B1R	GGCTGAGCTTCTctactcaatcatCTGAATCTATACCTA
nleE_IE6_B2F	gagtagAGAAGCTCAGCC
nleE_IE6_B2R	AGAAAGCTGGGTcagttcacgaaacaatgtg
nleE_IE6_ckF	AGAACATCCGTATTTGAGC
nleE_IE6_ckR	TCCGGCATGTAACTGGC
for clone check	
SK1	CCCGAATTCGGCACAAGCATAAGC
SK2	CCCGGATCCGTCTCGCCAGTATTCG
bfpA_ORF_F	CTATTTCAGAAGTAATGAGCGCA
bfpA_ORF_R	AAGTACCTAARTTCAAVGTTGCA
for fosmid end sequencing	
Fos-F	TCCCAGTCACGACGTTG
Fos-R	ACCATGATTACGCCAAGC
