# Supplementary figures and images for: NleC, a Type III Secretion Protease, Compromises NF-κB Activation by Targeting p65/RelA
Source: PLoS Pathog. 2010 Dec 16;6(12):e1001231. doi: 10.1371/journal.ppat.1001231 (PMC3002990; doi:10.1371/journal.ppat.1001231)

Supplemental Figure S1. Gene organizations of the inserted fragments in pTOK-01 and pTOK-02

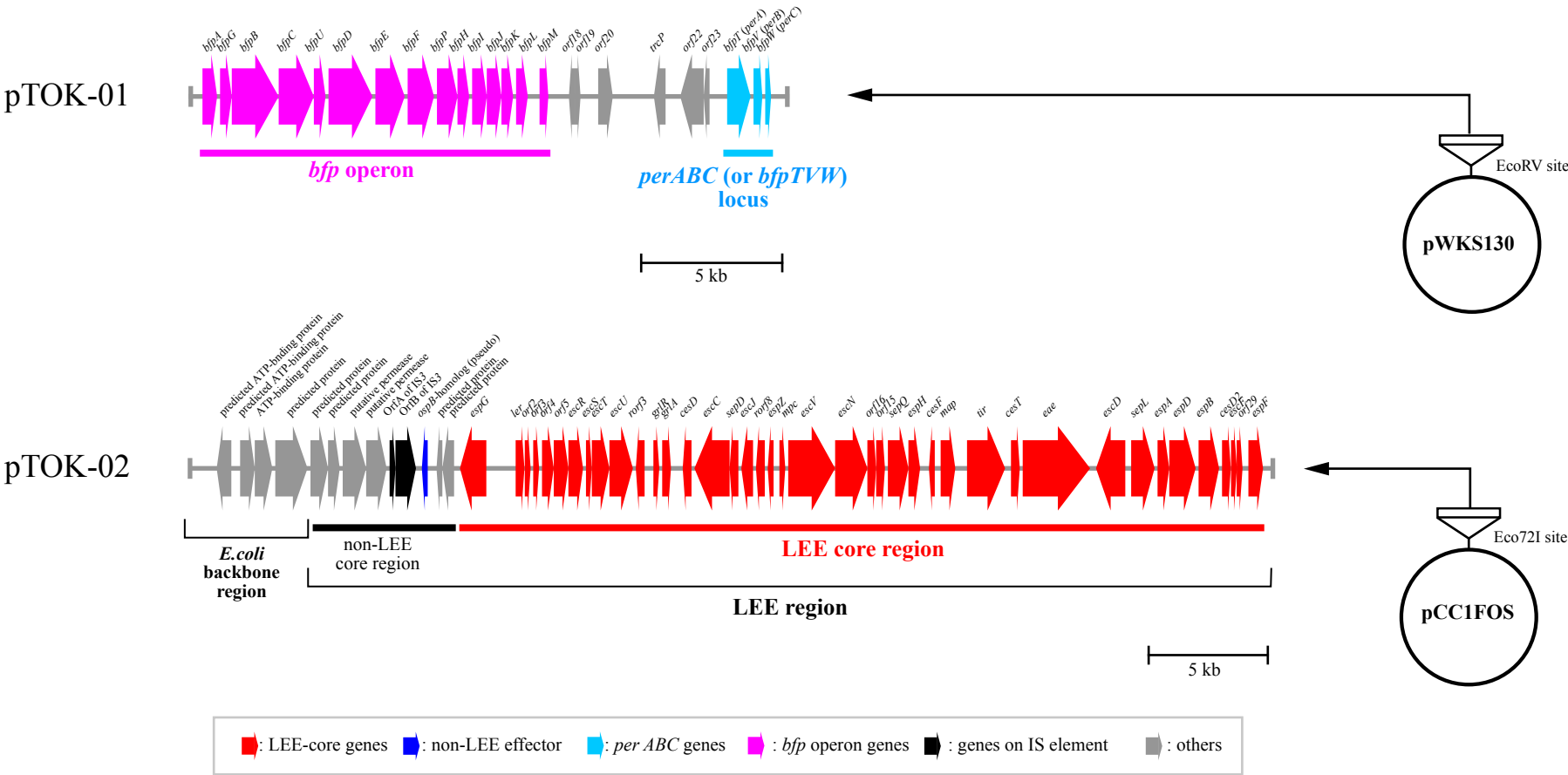

Supplement: Figure S1 — Gene organizations of the inserted fragments in pTOK-01 and pTOK-02. (0.73 MB PDF) [file ppat.1001231.s003.pdf]

**Supplemental Figure S2. Tir translocation between EPEC wild type,  $\Delta$ escF, and TOB02.**

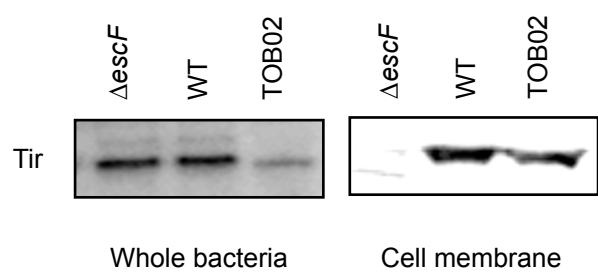

Supplement: Figure S2 — Comparison of Tir translocation efficiency between wild type EPEC and reconstructed strain TOB02. HeLa cells were infected by m.o.i of 100 of pre-activated wild type EPEC, ΔescF (T3SS defective mutant), and TOB02 for 2 hours. Cells were rinsed with cold PBS twice to remove the non-adherent bacteria, scrapped and collected in the presence of sonication buffer (50 mM NaCl, 50 mM Tris pH 7.9, 10% sucrose (w/v), 2 mM EDTA, 0.4 mM Na3VO4, 1× protease inhibitor cocktail). Samples were sonicated for 3 sec to disrupt mammalian but not bacterial membrane. Then ultracentrifugation was applied at 45,000 rpm, 15 min. at 4°C. The pellet was washed once with sonication buffer and resuspended in the lysis buffer (50 mM NaCl, 50 mM Tris pH 7.9, 10% sucrose (w/v), 2 mM EDTA, 0.4 mM Na3VO4, 0.5% Triton-X, and 1× protease inhibitor cocktail). Lysates were subjected to second round of ultracentrifugation (45,000 rpm, 5 min. at 4°C). Finally the supernatant which contained the membrane fraction (host cell membrane) was collected. For the bacterial whole cell (bacteria), the bacterial pellet from 1 ml of the activation culture was lysed with 2× SDS sampler buffer. Samples were analyzed by immunoblotting using anti-Tir antibody. This experiment was repeated three times and the representative blot is shown. (0.08 MB PDF) [file ppat.1001231.s004.pdf]

Supplemental Figure S3. Secretion assay of TOB02/*nleC*-HA

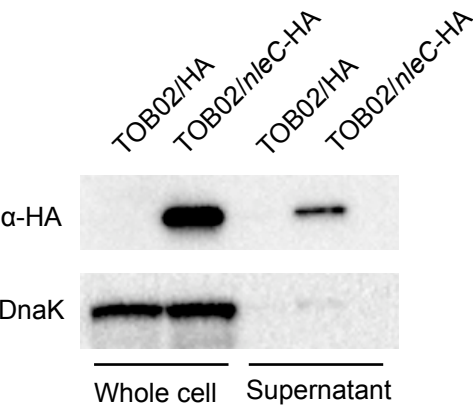

Supplement: Figure S3 — TOB02/nleC-HA is able to secret NleC-HA via a reconstituted T3SS. Overnight-grown TOB02/HA and TOB02/nleC-HA were inoculated into DMEM for the secretion assay. When the bacterial growth reached O.D600 ∼1.0, the supernatant and bacterial pellet were separated by centrifugation and subjected to immunoblot analysis. An anti-HA antibody was used to detect the expression of NleC-HA, and anti-DnaK was used to verify that the supernatant was free of whole-bacteria contaminants. (0.09 MB PDF) [file ppat.1001231.s005.pdf]

**Supplemental Figure S5. The degradation of p65-C is dependent on the proteasome**

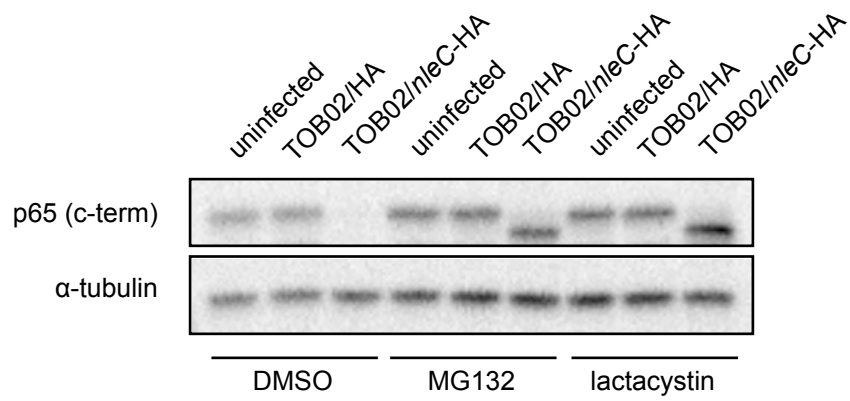

Supplement: Figure S5 — The degradation of p65-C is dependent on the proteosome. HeLa cells were first pretreated with either DMSO, or MG132 (5 µM), or lactacystin (10 µM) for 2 hours. Pre-activated TOB02/HA or TOB02/nleC-HA was used to infect the cells for another 2 hours. The cells were rinsed with PBS to remove non-adherent bacteria and lysed directly by addition of SDS sampler buffer. Samples were analyzed using anti-p65 (C-term) and anti-alpha-tubulin antibodies. (0.08 MB PDF) [file ppat.1001231.s007.pdf]

**Supplemental Figure S6. Cleavage of p65 is not due to apoptotic event during the infection**

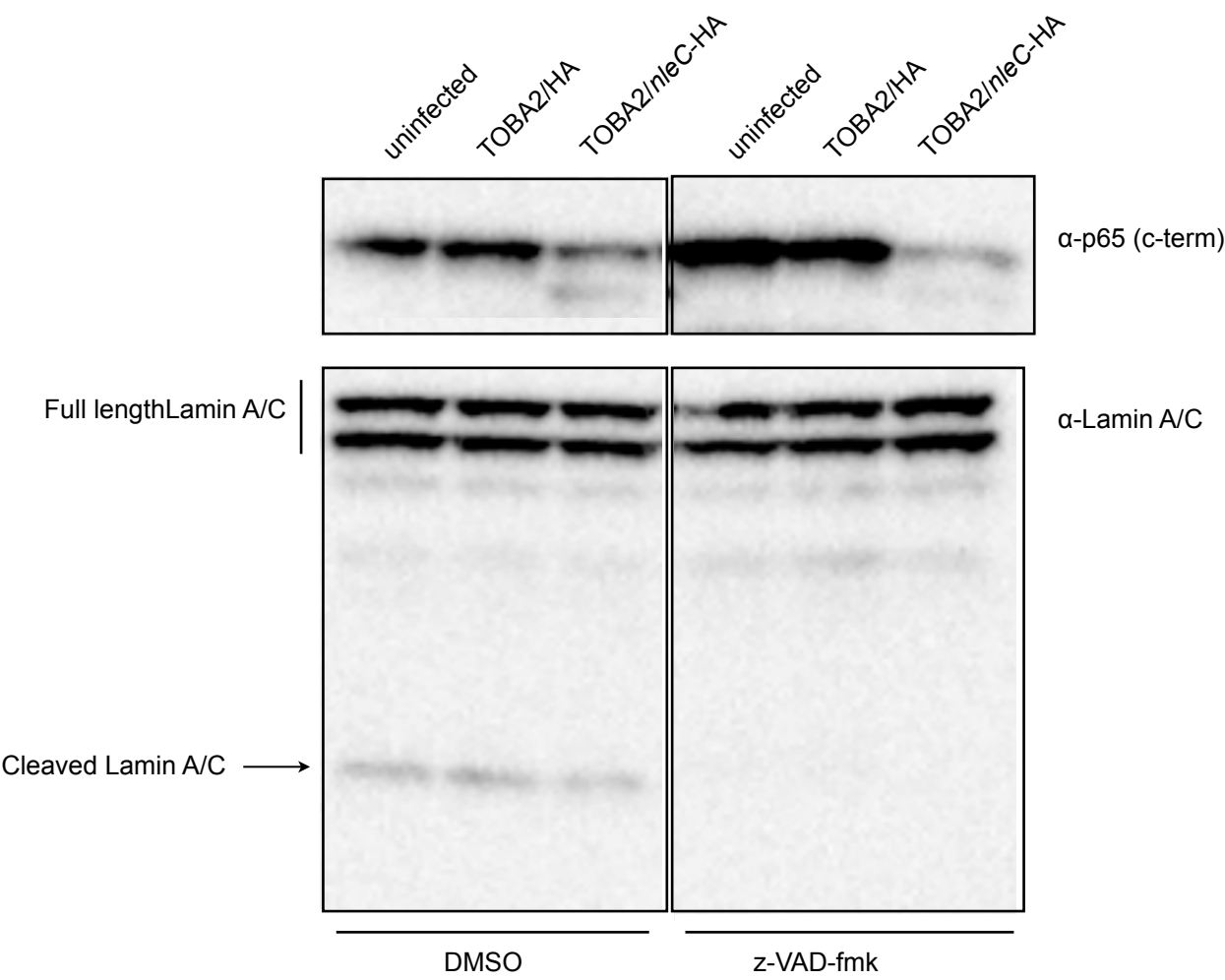

Supplement: Figure S6 — Caspase-3 is not involved in p65 cleavage during the TOBA2/nleC-HA bacterial infection. HeLa cells were first pretreated with either DMSO or z-VAD-fmk (5 µM) for 2 hours. Pre-activated TOB02/HA or TOB02/nleC-HA was then used to infect the cells for another 2 hours. The cells were rinsed with PBS to remove non-adherent bacteria and lysed by the direct addition of SDS sampler buffer. The cell samples were analyzed using anti-p65 (C-term.) and anti-Lamin A/C antibodies by immunoblotting. (0.10 MB PDF) [file ppat.1001231.s008.pdf]

**Supplemental Figure S7. Cleavage and generation of p65-C in WT EPEC**

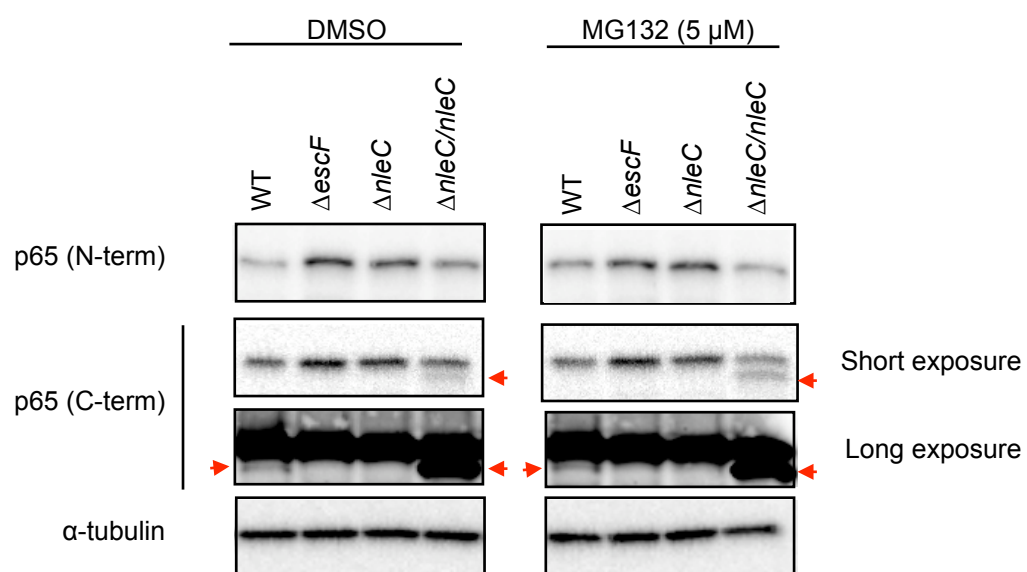

Supplement: Figure S7 — Generation of p65-C in native wild-type EPEC. HeLa cells pretreated with either DMSO or MG132 (5 µM) were infected with EPEC of wild type (WT), ΔescF, ΔnleC, and ΔnleC/nleC for 2 hours. Non-adherent bacteria were removed by washing cells with PBS. Cells were then directly lysed with sample buffer and analyzed by anti-p65 (N-term), anti-p65 (C-term), and α-tubulin (loading control) antibodies. Short and Long exposure of the membrane were taken. p65-C fragment is indicated by arrowheads. This experiment was repeated three times and one of the representative blot is shown. (0.11 MB PDF) [file ppat.1001231.s009.pdf]

**Supplemental Figure S8. NleH1 does not contribute significantly in suppression of IL-8 response**

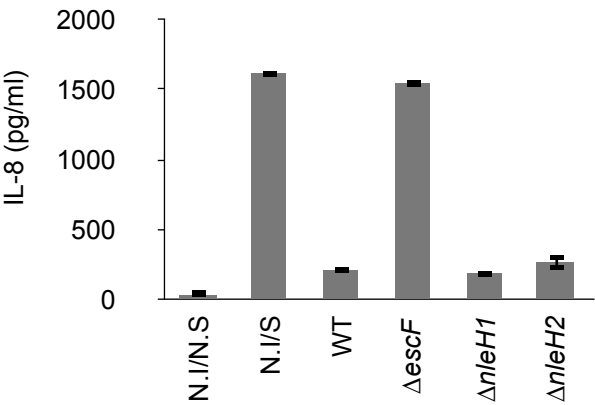

Supplement: Figure S8 — EHEC ΔnleH1 does not alleviate the immune suppression. HeLa cells were infected with pre-activated wild-type, or the ΔescF, ΔnleH1, and ΔnleH2 isogenic mutants of EHEC. After 4 hours of infection, the cell culture medium was replaced with fresh DMEM containing gentamicin (0.1 mg/ml) and HKE (1/10 vol.). The cells were further cultured for 8 hours, and the medium was then collected for IL-8 analysis by ELISA. Similar experiments were performed in triplicate and repeated three times. Student's t-test was used, and p<0.05 was considered significant. (0.04 MB PDF) [file ppat.1001231.s010.pdf]

Supplemental Figure S9. Association of p65-C with IκBα in cytoplasm

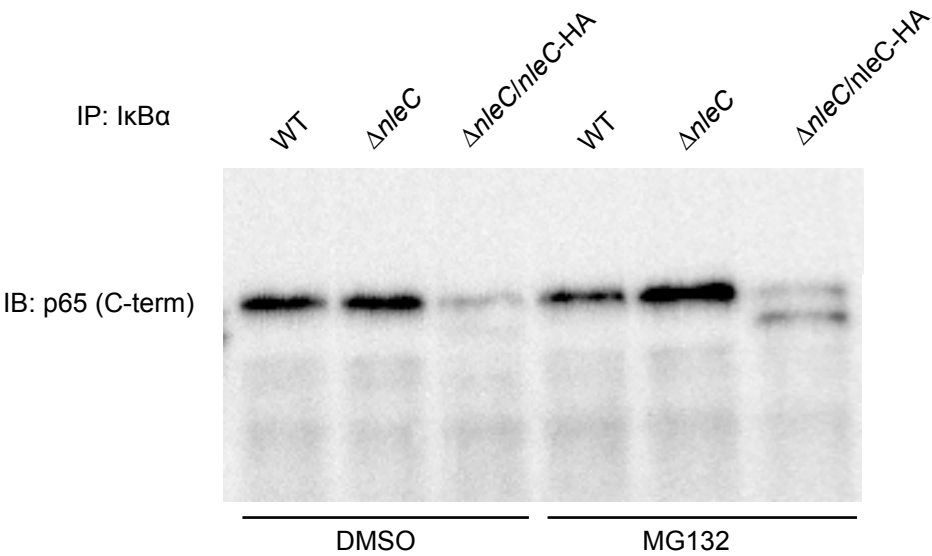

Supplement: Figure S9 — IκBα-associated p65 can be processed by NleC. HeLa cells pretreated with either DMSO or MG132 (5 µM) were infected with wild-type, ΔnleC, or ΔnleC/pnleC-HA of EPEC for 2 hours. At the end of infection, the HeLa cells were rinsed with PBS to remove non-adherent bacteria, and lysed with NET150 on ice. The cytoplasmic fraction was immunoprecipitated with an anti-IκBα antibody, and the pull-down products were analyzed using an anti-p65 (C-term.) antibody by immunoblotting. (0.09 MB PDF) [file ppat.1001231.s011.pdf]

**Supplemental Figure S10. In vitro cleavage assay of p50 and IκBα**

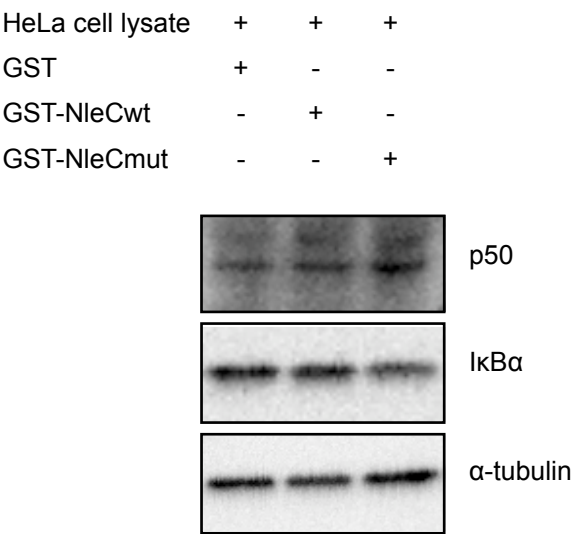

Supplement: Figure S10 — NleC does not cleave p50 nor IκB in vitro. Cell lysates were prepared from unstimulated HeLa cells. The lysates were then mixed with GST, GST-NleCwt, and GST-NleCmut in vitro and were incubated at 25°C for 8 hours. The reactions were terminated by direct addition of 2× sample buffer. Samples were analyzed by immunoblotting using anti-p50, anti-IκBα, and anti-tubulin antibodies. This experiment was repeated three times and the representative blot is shown. (0.07 MB PDF) [file ppat.1001231.s012.pdf]
