# Supplementary material for: NleC, a Type III Secretion Protease, Compromises NF-κB Activation by Targeting p65/RelA
Source: PLoS Pathog. 2010 Dec 16;6(12):e1001231. doi: 10.1371/journal.ppat.1001231 (PMC3002990; doi:10.1371/journal.ppat.1001231)

Supplemental Figure S4. Cytoplasmic localization of NleC by WT O157:H7 Sakai strain via endogenous T3SS

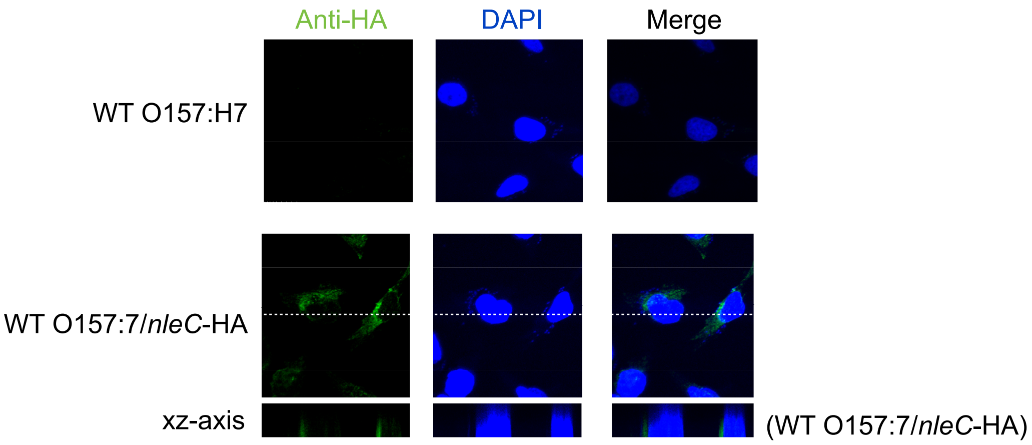

Supplement: Figure S4 — NleC-HA can be secreted by the endogenous T3SS in EHEC O157:H7, and localizes to the cytoplasm. The NleC-HA-expressing wild-type EHEC O157:H7 was used to infect HeLa cells. After 4 hours of infection, the cells were washed with PBS to remove non-adherent bacteria, and fixed with 4% PFA (paraformaldehyde). The cells were then stained with anti-HA (FITC) and with DAPI to label DNA. Photographs and the xz-axis views were taken using a Radiance 2100 confocal laser scanning microscope (Bio-Rad). (0.34 MB PDF) [file ppat.1001231.s006.pdf]
